# Crosslinking activity of non-muscle myosin II is not sufficient for embryonic cytokinesis in *C. elegans*

**DOI:** 10.1242/dev.179150

**Published:** 2019-11-01

**Authors:** Daniel S. Osório, Fung-Yi Chan, Joana Saramago, Joana Leite, Ana M. Silva, Ana F. Sobral, Reto Gassmann, Ana Xavier Carvalho

**Affiliations:** 1 Instituto de Investigação e Inovação em Saúde (i3S), Universidade do Porto, 4200-135 Porto, Portugal; 2 Instituto de Biologia Molecular e Celular, Universidade do Porto, 4200-135 Porto, Portugal

**Keywords:** *C. elegans*, Actomyosin contractility, Contractile ring, Cytokinesis, Non-muscle myosin II mutants, Muscle myosin II mutants

## Abstract

Cytokinesis in animal cells requires the assembly and constriction of a contractile actomyosin ring. Non-muscle myosin II is essential for cytokinesis, but the role of its motor activity remains unclear. Here, we examine cytokinesis in *C. elegans* embryos expressing non-muscle myosin motor mutants generated by genome editing. Two non-muscle motor-dead myosins capable of binding F-actin do not support cytokinesis in the one-cell embryo, and two partially motor-impaired myosins delay cytokinesis and render rings more sensitive to reduced myosin levels. Further analysis of myosin mutants suggests that it is myosin motor activity, and not the ability of myosin to crosslink F-actin, that drives the alignment and compaction of F-actin bundles during contractile ring assembly, and that myosin motor activity sets the pace of contractile ring constriction. We conclude that myosin motor activity is required at all stages of cytokinesis. Finally, characterization of the corresponding motor mutations in *C. elegans* major muscle myosin shows that motor activity is required for muscle contraction but is dispensable for F-actin organization in adult muscles.

This article has an associated ‘The people behind the papers’ interview.

## Abstract (precis)

**Highlighted Article:** The motor activity of non-muscle myosin II is essential for cytokinesis and contributes to all stages of the process in *C. elegans* embryos.

## INTRODUCTION

Cytokinesis is the final step of cell division that leads to the partitioning of the mother cell into two daughter cells, thereby ensuring that each daughter retains one copy of the replicated genome. Although cell-substrate adhesion may facilitate division in cultured cells (Dix et al., 2018; Kanada et al., 2005; Nagasaki et al., 2009; Neujahr et al., 1997), in fungi and animals cytokinesis primarily relies on the assembly and constriction of a distinct acto-myosin structure, the contractile ring, that forms at the cell equator. In animal cells, the contractile ring assembles beneath the plasma membrane after anaphase onset and subsequently constricts, folding the cell membrane inwards to achieve the physical separation between daughter cells (Green et al., 2012).

The major components of the contractile ring are filamentous actin (F-actin) and non-muscle myosin II (hereafter myosin). F-actin composes the scaffold of the contractile ring (Carvalho et al., 2009; Silva et al., 2016; Stachowiak et al., 2014; Wollrab et al., 2016) and F-actin dynamics in the ring are likely controlled by a variety of actin-binding proteins, including formins, profilin, cofilin, capping proteins and several crosslinkers such as α-actinin and fimbrin (Blanchoin et al., 2014).

Myosin is a motor protein that has traditionally been regarded as the engine that drives cytokinesis, but recent work has challenged this view (see below). Myosin is a hexameric complex composed of a dimer of heavy chains and two pairs of light chains. Each heavy chain has a N-terminal globular head that contains an ATP-binding pocket and an actin-binding site, a lever arm where the light chains bind, and a C-terminal coiled-coil domain involved in interactions that promote heavy chain dimerization and formation of multi-headed bipolar filaments (Niederman and Pollard, 1975; Vicente-Manzanares et al., 2009). ATP hydrolysis induces coupled conformational changes that are transmitted through the head subdomains to the lever arm. This generates a power stroke that causes myosin to move towards the actin filament barbed-end. In an interconnected F-actin network with antiparallel filament arrangement, this movement causes filaments to slide past one another and the network to contract. In addition, the ability to bind actin allows myosin filaments to exert tension and maintain the network connected. Ultrastructural studies show that the contractile ring consists primarily of unbranched filaments aligned parallel to the ring circumference and arranged in an antiparallel manner (Henson et al., 2017; Kamasaki et al., 2007; Maupin and Pollard, 1986; Sanger and Sanger, 1980; Schroeder, 1973). Additionally, myosin has been shown to form arrays of aligned filaments or stacks of filaments running parallel to actin filaments, an organization that is compatible with a purse-string mechanism where F-actin sliding by myosin motors would drive ring constriction (Beach et al., 2014; Fenix et al., 2016; Henson et al., 2017). However, although myosin is essential for cytokinesis in different model systems (De Lozanne and Spudich, 1987; Mabuchi and Okuno, 1977; Straight et al., 2003), the specific requirement for myosin motor activity has been a subject of recent debate. Budding yeast is able to perform cytokinesis in the presence of a motor-less myosin (Lord et al., 2005; Mendes Pinto et al., 2012). In fission yeast, myosin motor activity appears to be required for ring constriction, but additional myosins also contribute (Laplante and Pollard, 2017; Laplante et al., 2015; Palani et al., 2017). In the amoeba *Dictyostelium discoideum*, specific mutations within the ATPase domain result in motor-dead myosins that, when expressed in suspension cells, cause growth phenotypes similar to or more severe than those of cells expressing no myosin, highlighting the importance of motor activity in this system (Sasaki et al., 1998; Shimada et al., 1997). Whether myosin motor activity is absolutely required in animal cells is less clear, as studies have relied on the use of the small molecule inhibitor blebbistatin, depletion/inactivation of myosin/myosin temperature-sensitive mutants or depletion or non-phosphorylatable mutants of the regulatory light chain that is required for myosin complex activation (Davies et al., 2014; Descovich et al., 2018; Jordan and Karess, 1997; Reymann et al., 2016; Straight et al., 2003). None of these approaches can provide a definitive answer regarding the requirement for motor activity, as blebbistatin keeps myosin in a low actin affinity state (Allingham et al., 2005), depletion or inactivation of myosin does not differentiate between motor activity and F-actin crosslinking, and interfering with the regulatory light chain may affect other myosins or influence myosin localization and/or structure (Heissler and Sellers, 2015; Liu et al., 2016; Vasquez et al., 2014). The effect of specific motor-impairing mutations has been reported for the mammalian myosin IIB in COS-7 cells and mouse cardiomyocytes (Ma et al., 2012), and in this case the requirement for myosin motor activity was contested*.* Thus, how the contractile ring produces the force to form the cytokinetic furrow remains an important question.

The *C. elegans* embryo is particularly suited for the quantitative *in vivo* analysis of cytokinesis, as the embryo is large and its divisions are stereotypical and temporally invariant. *C. elegans* possesses two non-muscle myosin II heavy chains: NMY-1 and NMY-2. NMY-2 has been shown to be essential for cytokinesis (Cuenca et al., 2003; Davies et al., 2014; Guo and Kemphues, 1996), whereas NMY-1 is required during late embryonic development (Piekny et al., 2003), in the adult somatic gonad and the spermatheca (Kovacevic et al., 2013; Priti et al., 2018; Wirshing and Cram, 2017). In this study, we assess the role of myosin motor activity during cytokinesis in the *C. elegans* early embryo by characterizing NMY-2 motor mutants generated by genome editing. Our results suggest that it is myosin motor activity, and not the ability of myosin to crosslink F-actin, that drives ring assembly by compacting and aligning F-actin bundles. Furthermore, we find that myosin motor activity determines the pace of constriction.

## RESULTS

### Expression of motor-dead muscle myosins prevents *C. elegans* locomotion without substantially affecting actin organization in body wall muscles

**Fig. 1.**
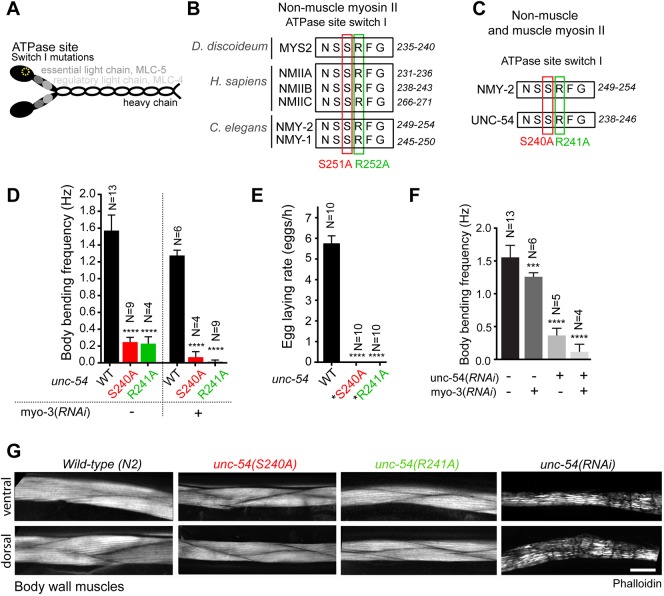
**Muscle contraction as an *in vivo* readout of motor-impairment in myosin II mutants.** (A) Schematic of the non-muscle myosin II hexamer. (B) Alignment of non-muscle myosin II sequences. The highly conserved residues S251 and R252A (numbered as in *C. elegans* NMY-2) mutated to alanine to obtain putative motor-dead myosins are marked by red and green boxes, respectively. (C) Residues S240 and R241 (numbered as in *C. elegans* UNC-54) mutated to alanine to obtain putative motor-dead muscle myosin are marked by red and green boxes, respectively. (D) Body bend frequency in liquid (mean±95% CI) in wild-type and *unc-54* motor mutant animals with and without depletion of the secondary muscle myosin MYO-3. (E) Egg-laying rate (mean±95% CI) in wild-type and *unc-54* mutant animals. Mutant animals do not lay eggs, but embryos are viable and develop normally inside the mother (asterisk). (F) Body bend frequency in liquid (mean±95% CI) in wild-type animals depleted of UNC-54 or MYO-3, or both. N is the number of analyzed animals in D, E and F. (G) Dorsal and ventral views of phalloidin-stained body wall muscles in animals with indicated genotypes and RNAi treatments. Statistical significance was determined using one-way ANOVA followed by Bonferroni's multiple comparison test; ****P≤0.0001, ***P≤0.001. Scale bar: 10 μm.

To generate motor-dead myosin mutants in *C. elegans*, we took advantage of a previous alanine mutagenesis screen in the highly conserved switch I region of the ATPase domain of *D. discoideum* non-muscle myosin II, which yielded a series of mutants with compromised motor activity (Shimada et al., 1997). Based on the high sequence conservation among myosins, we chose two point mutations shown to yield motor-dead myosin in *D. discoideum*. These mutations correspond to S251A and R252A in NMY-2, and to S240A and R241A in UNC-54, the main skeletal muscle myosin that is essential for animal movement and egg laying (Epstein and Thomson, 1974; Fire et al., 1991; Fig. 1A-C, Fig. S1).

To assess the potential of these mutations to affect myosin motor activity in *C. elegans*, we first examined the consequences of introducing them into muscle myosin. Muscle fibers are composed of sarcomeres, which require myosin motor activity to contract. Although muscle and non-muscle myosin II present differences in ATP hydrolysis kinetics and motility rates, the principle underlying the change in molecular conformation that allows for the power stroke is identical in both motors and relies on extremely well-conserved regions, including the switch I loop of the ATP-binding site (Heissler and Sellers, 2016; Fig. 1B,C, Fig. S1). Moreover, previous studies have established that the mechanistic effects of mutations in conserved myosin head residues are transposable between class II myosins and even between different myosin classes (Forgacs et al., 2009; Li et al., 1998; Onishi et al., 1998; Trivedi et al., 2012).

Using CRISPR/Cas9-based genome editing (Arribere et al., 2014), we introduced the two point mutations into UNC-54. We were able to generate homozygous animals expressing UNC-54(S240A) and UNC-54(R241A). To evaluate muscle function, we monitored animal locomotion and egg laying rates (Fig. 1D,E). *unc-54(S240A)* and *unc-54(R241A)* adult animals displayed a drastic reduction in liquid locomotion [0.24±0.04 Hz in *unc-54(S240A)*, 0.23±0.13 Hz in *unc-54(S241A)* versus 1.6±0.1 Hz in wild-type animals] and were unable to lay eggs, as expected for strongly motor-impaired myosins. Residual movement observed in *unc-54(S240A)* and *unc-54(R241A)* animals was attributable to the secondary body wall muscle myosin MYO-3, as depletion of MYO-3 in either *unc-54* mutant led to paralysis on food plates and loss of motility in liquid (Fig. 1D). Penetrant loss of movement was also observed when UNC-54 and MYO-3 were co-depleted by RNAi in wild-type animals (Fig. 1F). As no neuronal roles have been described for UNC-54, it is reasonable to assume that decreased movement in *unc-54* animals is due to impairment of muscle contraction.

Interestingly, phalloidin staining of muscles in *unc-54(S240A)* and *unc-54(R241A)* adult animals revealed that actin organization was preserved (Fig. 1G). This is in agreement with reports of nearly normal sarcomere organization and substantially decreased ability to move in *unc-54(s74)* animals, which express a point mutation in the myosin head domain (R270C) (Hwang et al., 2016; Moerman et al., 1982). In contrast, depletion of UNC-54 by RNAi resulted in wavy and irregular F-actin bundles (Fig. 1G). This suggests that the motor activity of UNC-54 is not required for actin organization in adult muscles but is required for muscle contraction.

We conclude that mutating the highly conserved residues S240 and R241 results in inactive UNC-54 *in vivo*, in agreement with the effects of the mutations on non-muscle myosin II motor activity reported *in vitro* for *D. discoideum*. Of note, we also tested the mutation corresponding to R709C in mammalian non-muscle myosin IIB (Fig. S5A), a disease-related mutation in the SH1 helix (Ma et al., 2012). We found that muscle function was only partially impaired in *unc-54(R710C)* animals (Fig. S5B,C). UNC-54(R710C) is therefore unlikely to be motor-dead.

### NMY-2(S251A) and NMY-2(R252A) bind but do not translocate F-actin *in vitro*

**Fig. 2.**
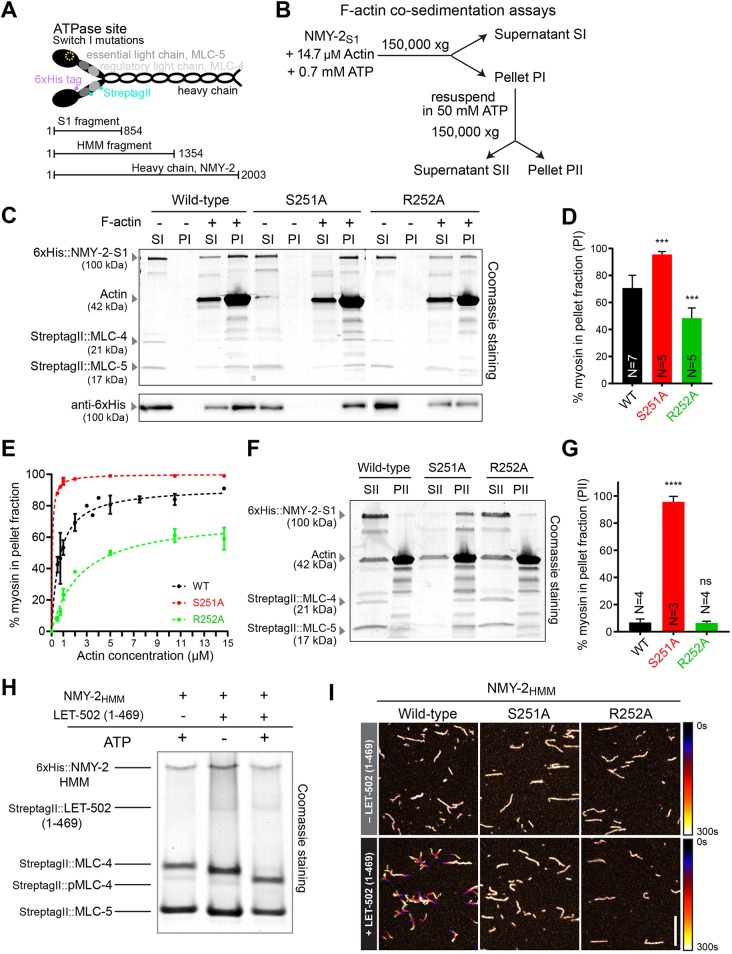
**NMY-2(S251A) and NMY-2(R252A) bind but are unable to translocate actin filaments *in vitro*.** (A) Schematic of the non-muscle myosin II depicting tags used for protein purification and S1 and HMM fragments. (B) Experimental procedure followed in C-G. (C) Coomassie-stained SDS-PAGE gel of SI and PI fractions from high-speed F-actin co-sedimentation assays (top). Immunoblot using an antibody against the 6xHistidine-tag (bottom). (D) Mean percentage±95% CI of NMY-2 S1 present in PI, determined by measuring protein band intensities in Coomassie-stained SDS-PAGE gels as shown in C. (E) Mean percentage±s.d. of NMY-2 S1 present in the pellet as a function of actin concentration. Dashed lines indicate the fitting of a one-site specific binding model using least-squares non-linear regression. (F) Coomassie-stained SDS-PAGE gel of SII and PII fractions from high-speed F-actin co-sedimentation assays. (G) Mean percentage±95% CI of NMY-2 S1 present in PII. (H) Coomassie-stained native gel of NMY-2_HMM_ incubated with ATP, LET-502(1-469) or ATP and LET-502(1-469). (I) Time projections of selected regions of Movie 1 showing F-actin sliding in the presence of wild-type or mutant NMY-2_HMM_ in the absence or presence of LET-502(1-469). Color coding was used from black (0 s) to white (300 s). N is the number of independent experiments in D and G. Statistical significance was determined using one-way ANOVA followed by Bonferroni's multiple comparison test; ****P≤0.00001; ***P≤0.001; ns, not significant (*P*>0.05). Scale bar: 10 μm.

We next characterized the ability of corresponding NMY-2 mutants to bind and translocate F-actin *in vitro*. First, we assessed F-actin binding of NMY-2(S251A) and NMY-2(R252A) in high-speed co-sedimentation assays. His-tagged NMY-2 S1 fragments (residues 1-854; routinely used for actin binding and kinetic assays; Manstein et al., 1989) carrying either mutation were purified along with the myosin regulatory (MLC-4) and essential (MLC-5) light chains from baculovirus-infected insect cells (hereafter NMY-2_S1_; Fig. 2A). NMY-2_S1_ was incubated with or without F-actin in the presence of 0.7 mM ATP before ultracentrifugation (Fig. 2B-D). In the absence of F-actin, all myosin was present in the supernatant (SI), indicating that wild-type and mutant NMY-2_S1_ are equally soluble. Conversely, in the presence of F-actin, all NMY-2_S1_ versions were found in the pellet (PI), showing that the mutants are capable of binding F-actin (Fig. 2C,D). F-actin pelleted in the absence of myosin as expected, and no proteins corresponding to the size of the NMY-2 S1 fragment were present in the pelleted fraction (Fig. S2A). The identity of NMY-2_S1_ was confirmed by immunoblotting (Fig. 2C). More of NMY-2(S251A)_S1_ and less of NMY-2(R252A)_S1_ pelleted with F-actin compared with wild-type NMY-2_S1_ (Fig. 2C,D). Specifically, by performing the pelleting assay at different actin concentrations, we obtained a Kd of 0.062±0.016 µM, 2.46±0.69 µM and 0.66±0.13 µM for NMY-2(S251A)_S1_, NMY-2(R252A)_S1_ and wild-type NMY-2_S1_, respectively (Fig. 2E). As a significant fraction of the ATP present in the buffer may be consumed during the course of the assay (thereby increasing the affinity of myosin for F-actin), the differences in Kd may in part reflect differences in the kinetics of ATP hydrolysis between wild-type and mutant myosins.

To determine whether the NMY-2 mutants were able to cycle between the F-actin bound and unbound states, we tested their ability to detach from F-actin pellets (PI) in the presence of high ATP concentrations. Myosin affinity for F-actin is determined by the status of the nucleotide bound to the ATPase pocket and is weak when bound to ATP (Spudich, 2001). PI pellets were resuspended in buffer containing 50 mM ATP in order to maintain myosin saturation (Fig. 2B). After ultracentrifugation, the supernatant (SII) and pellet (PII) were analyzed (Fig. 2F,G). Both wild type and NMY-2(R252A)_S1_ were almost completely displaced from PII, indicating that myosin detached from F-actin due to the high ATP concentration. NMY-2(S251A)_S1_ remained in PII, indicating that it either did not detach or was able to rebind F-actin even in the presence of high ATP.

To assess the ability of the NMY-2 mutants to translocate F-actin, we performed *in vitro* motility assays. Heavy meromyosin (HMM) fragments (residues 1-1354 based on Hu et al., 2002; Fig. 2A), which are better suited than S1 fragments for this type of assay, were purified together with MLC-4 and MLC-5 (hereafter NMY-2_HMM_; Fig. S2B). Activation of myosin contractility requires phosphorylation of its regulatory light chain and motility assays commonly use the calmodulin-dependent myosin light chain kinase (MLCK) for myosin activation (Sellers, 1998). However, this pathway does not seem to be required for embryonic cytokinesis in *C. elegans* (Batchelder et al., 2007), which is more dependent on the RhoA kinase LET-502 (Piekny and Mains, 2002). We produced a truncated version of this kinase, LET-502(1-469), which is homologous to the human ROCK1 minimal kinase domain (residues 3-415) previously shown to be active *in vitro* (Khandekar et al., 2006). Incubation of NMY-2_HMM_ with LET-502(1-469) and ATP led to a shift of the MLC-4 band in a native gel (Fig. 2H). Analysis of the shifted band by mass spectrometry confirmed that it corresponded to MLC-4 phosphorylated on residue S17 or T18 (not distinguishable). LET-502(1-469)-activated NMY-2_HMM_ was attached to the bottom of a flow chamber and rhodamine-labeled F-actin was flowed into the chamber. Imaging of F-actin demonstrated that wild-type NMY-2_HMM_ was able to translocate F-actin. In contrast, F-actin did not move in the presence of NMY-2(S251A)_HMM_ or NMY-2(R252A)_HMM_. In the absence of kinase, all NMY-2_HMM_ versions bound to F-actin but filaments remained mostly immobile (Fig. 2I, Movie 1).

In summary, these results show that wild-type NMY-2_HMM_ is capable of both binding and sliding F-actin *in vitro*, whereas NMY-2(S251A)_HMM_ and NMY-2(R252A)_HMM_ bind to but do not slide F-actin. While NMY-2(R252A)_S1_ appears to be able to cycle on and off F-actin similar to wild-type myosin, NMY-2(S251A)_S1_ is ATP insensitive and locked in a high actin-affinity conformation.

### Myosin motor activity is essential for embryo production and development

**Fig. 3.**
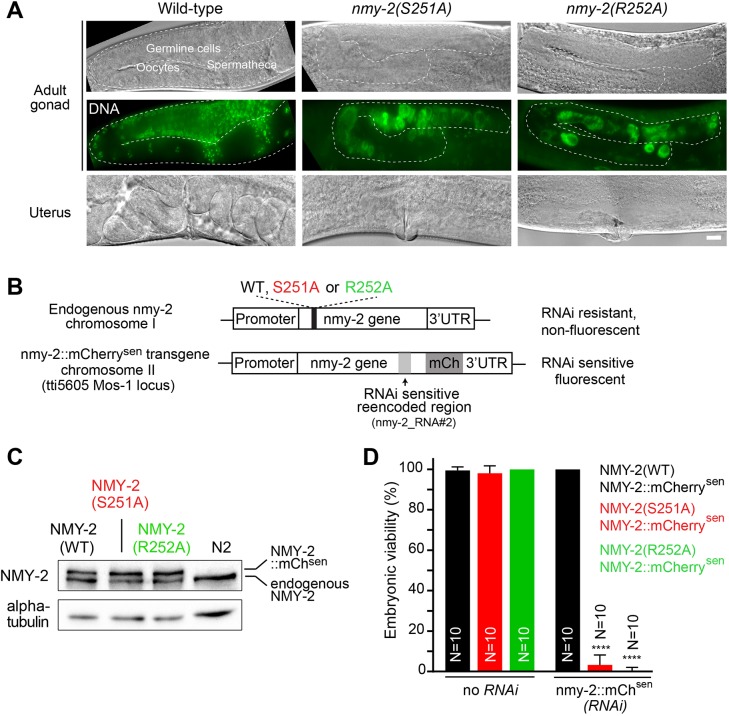
**Motor-dead non-muscle myosin II does not support embryonic development.** (A) Differential interference contrast images of the gonad (top) or uterus (bottom) in adult animals with indicated genotypes. Fluorescence images of DAPI-labeled DNA in the gonad region are shown in the central row. (B) Schematic of the endogenous and transgenic *nmy-2* loci. Mutations were introduced in the endogenous *nmy-2* gene on chromosome I by CRISPR/Cas9-mediated genome editing. A wild-type transgenic version of *nmy-2* carrying a re-encoded region for RNAi sensitivity (sen) and fused to *mCherry* was introduced in single copy on chromosome II using MosSCI. (C) Immunoblot showing protein levels of endogenous NMY-2 and transgene-encoded NMY-2::mCherry^sen^ in wild-type and mutant animals. α-Tubulin is used as loading control. (D) Embryonic viability (mean±95% CI) in the strains shown in B with or without penetrant depletion of NMY-2::mCherry^sen^. N denotes the number of animals whose embryonic progeny was examined. Statistical significance was determined using one-way ANOVA followed by Bonferroni's multiple comparison test; ****P≤0.0001. Scale bar: 10 µm.

Having established that NMY-2(S251A) and NMY-2(R252A) are motor-dead *in vitro*, we next used genome editing to introduce the mutations into NMY-2 *in vivo* (Arribere et al., 2014). Animals homozygous for either mutation exhibited severe gonad malformation and were consequently sterile (Fig. 3A). To examine the impact of these mutants on embryogenesis, we introduced transgene-encoded wild-type NMY-2::mCherry into *nmy-2(S251A)* and *nmy-2(R252A)* animals. NMY-2::mCherry was expressed from the *nmy-2* promoter and 3′UTR and the transgene was partially re-encoded so it could be specifically depleted by RNAi (NMY-2::mCherry^sen^, sen indicating RNAi-sensitive; Fig. 3B). The resulting strains were homozygous for both versions of NMY-2, which were expressed at similar levels (Fig. 3C). The presence of NMY-2::mCherry^sen^ allowed homozygous *nmy-2(S251A)* and *nmy-2(R252A)* mutants to develop normally into adulthood and lay viable eggs. When NMY-2::mCherry^sen^ was penetrantly depleted using stringent RNAi conditions (see methods), *nmy-2(S251A)* and *nmy-2(R252A)* embryos were inviable (Fig. 3D). These results demonstrate that NMY-2 motor activity is required for embryonic development.

### Motor-dead myosins do not support cytokinesis

**Fig. 4.**
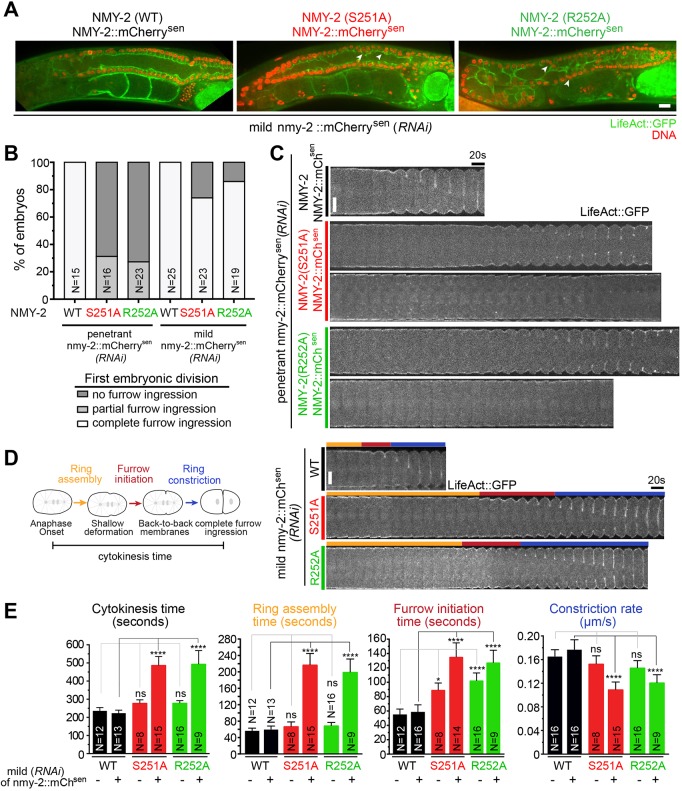
**Motor activity of non-muscle myosin II is essential for cytokinesis.** (A) Gonads of adult hermaphrodites with indicated genotypes. LifeAct::GFP labels gonad compartments and DNA is labeled with Hoechst 33342. Arrowheads indicate multinucleated compartments. (B) Percentage of embryos that complete (white), fail with partial ingression (light gray) or fail without ingression (dark gray) the first embryonic cytokinesis in animals with indicated genotypes after penetrant or mild depletion of NMY-2::mCherry^sen^. (C,D) Kymographs of the equatorial region of embryos with indicated genotypes after penetrant (C) or mild (D) depletion of NMY-2::mCherry^sen^. In C, two examples are shown for each mutant, one with partial furrow ingression and one with no furrow ingression. First frame corresponds to anaphase onset. Orange, red and blue bars indicate the intervals of ring assembly, furrow initiation and ring constriction, respectively, as depicted on the left in D. (E) Cytokinesis, ring assembly and furrow initiation time intervals and rate of ring constriction (mean±95% CI) in wild-type or mutant embryos subjected or not to mild depletion of NMY-2::mCherry^sen^. N is the number of embryos analyzed. Statistical significance was determined using one-way ANOVA followed by Bonferroni's multiple comparison test; ****P≤0.0001; *P≤0.05; ns, not significant (*P*>0.05). Scale bars: 10 µm.

Next, we asked whether NMY-2(S251A) and NMY-2(R252A) could support cytokinesis. Expression of the motor-dead myosins did not prevent wild-type NMY-2::mCherry^sen^ from localizing in cortical patches during the first embryonic cytokinesis. However, myosin patches outside the cell equator were less abundant than in controls (Movie 2). The time of cytokinesis was not significantly affected (Fig. 4E, -RNAi). When NMY-2::mCherry^sen^ was penetrantly depleted by RNAi, *nmy-2(S251A)* and *nmy-2(R252A)* animals were sterile with non-compartmentalized gonads (data not shown). When we used less stringent RNAi conditions (mild NMY-2::mCherry^sen^ depletion, see Materials and methods), the gonads of these animals presented multinucleated compartments, indicating problems with cytokinesis in this tissue (Fig. 4A).

Although *nmy-2(S251A)* and *nmy-2(R252A)* animals became sterile after penetrant depletion of NMY-2::mCherry^sen^, we were able to examine the last fertilized one-cell embryos produced before onset of sterility. The majority of these embryos failed to initiate furrow ingression [69% in *nmy-2(S251A)*; 74% in *nmy-2(R252A)*; Fig. 4B,C]. In the remaining embryos, the furrows ingressed partially, likely owing to the presence of residual wild-type NMY-2::mCherry^sen^ (Fig. 4B,C). We conclude that myosin motor activity is essential for cytokinesis.

To understand whether NMY-2(S251A) or NMY-2(R252A) affected a particular stage of cytokinesis, we performed mild depletions of NMY-2::mCherry^sen^. Under these conditions, the majority of mutant one-cell embryos completed cytokinesis but did so more slowly than wild-type embryos (490±45 s for S251A, 494±87 s for R252A, 219±14 s for controls; Fig. 4D,E). We defined two intervals during early cytokinesis based on easily identifiable reference points: (1) the interval between anaphase onset and the formation of a shallow equatorial deformation, when contractile ring components are being recruited to the cell equator (‘ring assembly’); and (2) the interval between shallow deformation and the folding of the plasma membrane into a back-to-back configuration (‘furrow initiation’) (Fig. 4D). To evaluate ring constriction, the rate of ring diameter decrease was calculated (Fig. 4E). Abscission in *C. elegans* embryos only completes in the following round of cell divisions and was not analyzed (Green et al., 2013). Mild depletion of NMY-2::mCherry^sen^ in embryos expressing motor-dead myosins increased both intervals and slowed the ring constriction rate (0.11±0.01 µm/s for S251A, 0.12±0.01 µm/s for R252A, 0.18±0.01 µm/s for controls; Fig. 4D,E). The effects on cytokinesis were comparable with those obtained after partial depletion of NMY-2 in otherwise wild-type embryos (Fig. S3A-C). Interestingly, furrow initiation was already slightly delayed in *nmy-2(S251A)* and *nmy-2(R252A)* embryos without depleting NMY-2::mCherry^sen^, suggesting that the presence of motor-dead myosin interfered with the function of the wild-type version (-RNAi, Fig. 4E).

Together, these data show that cytokinesis is progressively affected as the ratio of motor-dead to wild-type myosin increases. We conclude that myosin motor activity is required for ring assembly, furrow initiation and ring constriction, and that its ability to crosslink F-actin is therefore not sufficient for cytokinesis.

### Partial impairment of myosin motor activity slows ring constriction and reduces the robustness of cytokinesis

We generated animals expressing NMY-2(S250A), which is predicted to result in a partially motor-impaired myosin (Shimada et al., 1997). Like S251 and R252, the S250 residue is located in the switch I region of the ATPase domain of myosin (Fig. 1B, Fig. S1). We were able to generate homozygous animals expressing the equivalent mutation in muscle myosin: *unc-54(S239A)* (Fig. S4A-C). These animals displayed reduced locomotion in liquid (0.6±0.1 Hz versus 1.6±0.1 Hz in controls) and reduced egg-laying rate (3.7±0.5 eggs/h versus 5.8±0.3 eggs/h in controls), as expected for a myosin with partially-impaired motor activity (Fig. S4A,B). Body muscles in *unc-54(S239A)* animals presented a well-organized actin structure (Fig. S4C).

**Fig. 5.**
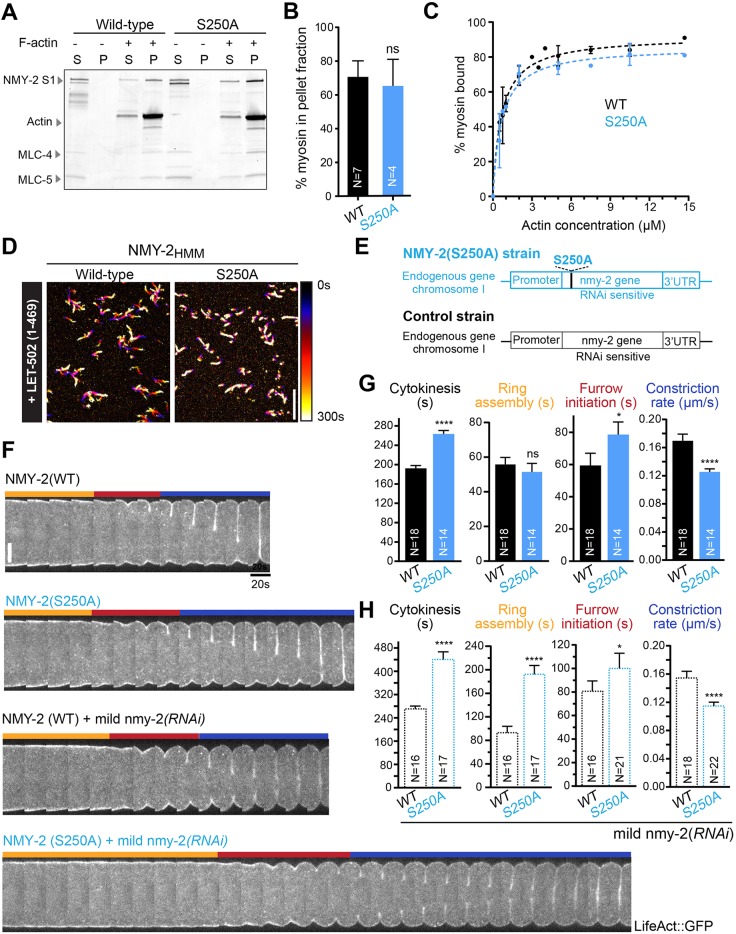
**Partial impairment of non-muscle myosin II motor activity slows ring constriction and reduces cytokinesis robustness.** (A) Coomassie-stained SDS-PAGE gel of supernatant (S) and pellet (P) fractions from high-speed F-actin co-sedimentation assays, where wild-type NMY-2_S1_ or NMY-2(S250A)_S1_ were incubated with 14.7 µM F-actin and 0.7 mM ATP before ultracentrifugation. (B) Mean percentage±95% CI of NMY-2 S1 present in the pellet, determined by measuring protein band intensities in Coomassie-stained SDS-PAGE gels as shown in A. (C) Mean percentage±s.d. of NMY-2 S1 present in the pellet as a function of actin concentration. Dashed lines indicate the fitting of a one-site specific binding model using least-squares non-linear regression. (D) Time projections of movies of F-actin sliding in the presence of NMY-2_HMM_ or NMY-2(S250A)_HMM_ after phosphorylation by LET-502(1-469). Color coding was used from black (0s) to white (300s). (E) Schematic of the *nmy-2* locus after introduction of the S250A mutation by CRISPR/Cas9-mediated genome editing. (F) Kymographs of the equatorial region of wild-type or S250A embryos with and without mild depletion of NMY-2. First frame corresponds to anaphase onset. Orange, red and blue bars indicate the intervals of ring assembly, furrow initiation and ring constriction, respectively, as depicted on the left in Fig. 4D. (G,H) Cytokinesis, ring assembly and furrow initiation time intervals and rate of ring constriction (mean±95% CI) in wild-type or S250A embryos without (G) or with (H) mild NMY-2 depletion. N is the number of independent experiments in B, and the number of analyzed embryos in G,H. Statistical significance was determined using one-way ANOVA followed by Bonferroni's multiple comparison test; ****P≤0.0001; *P≤0.05; ns, not significant (*P*>0.05). Scale bars: 10 µm.

NMY-2(S250A)_S1_ co-sedimented with F-actin (Fig. 5A,B), and its affinity for F-actin was similar to that of wild-type NMY-2_S1_ (0.66±0.13 µM for S250A and 0.66±0.21 µM for wild type). NMY-2(S250A)_HMM_ was able to slide F-actin but movement was reduced compared with controls (Fig. 5D, Fig. S4F).

Homozygous animals expressed NMY-2(S250A) at levels comparable with wild-type controls and were fully viable (Fig. S4D,E). Embryos expressing NMY-2(S250A) presented prolonged cytokinesis (263±7 s for S250A, 192±6 s for controls; Fig. 5G, Movie 3). Ring assembly time was normal but furrow initiation was delayed and ring constriction rate was decreased (0.12±0.01 µm/s for S250A and 0.17±0.01 µm/s for controls; Fig. 5G). We also generated NMY-2(R718C), which corresponds to R709C in mammalian myosin IIB (Fig. S5A). This mutant was able to bind F-actin similarly to wild type (Fig. S5D,E). Homozygous *nmy-2(R718C)* animals were fully viable and propagated normally (Fig. S5F), and analysis of cytokinesis revealed delays similar to those observed in *nmy-2(S250A)* embryos (Fig. S5G). Together with the partial impairment of muscle function when the equivalent mutation was introduced into UNC-54 (Fig. S5B,C), these results are consistent with the idea that this mutation compromises, but does not abolish, myosin motor activity.

Next, we asked whether embryos expressing wild-type, NMY-2(S250A) or NMY-2(R718C) were sensitive to a decrease in overall myosin levels. After mild depletion of endogenous wild-type or mutant NMY-2, most embryos completed cytokinesis, albeit more slowly than non-depleted controls (441±26 s for S250A, 290±25 s for R718C, 271±9 s for wild type and 263±7 s for S250A no RNAi, 262±9 s for R718C no RNAi, 192±6 s for WT no RNAi; Fig. 5F,H, Fig. S5G,H). Embryos with reduced levels of wild-type NMY-2 exhibited slight delays in ring assembly and furrow initiation, and a slight decrease in constriction rate (0.15±0.01 µm/s; Fig. 5H). In contrast, embryos with reduced levels of NMY-2(S250A) exhibited substantial delays in ring assembly and furrow initiation and a substantial decrease in ring constriction rate (0.11±0.01 µm/s; Fig. 5H). Additionally, four out of 22 *nmy-2(S250A)* embryos mildly depleted of NMY-2 failed cytokinesis, whereas all wild-type embryos completed cytokinesis successfully (18 out of 18). Mild depletion of NMY-2 in *nmy-2(R718C)* embryos decreased the ring constriction rate (0.13±0.01 µm/s; Fig. S5H) but did not affect the initial stages of cytokinesis nor lead to cytokinesis failure (Fig. S5H). We conclude that contractile rings with partially motor-impaired NMY-2 are less resilient to a decrease in myosin levels than rings with wild-type NMY-2.

### Perturbation of myosin levels or motor activity does not change actin levels during ring constriction

**Fig. 6.**
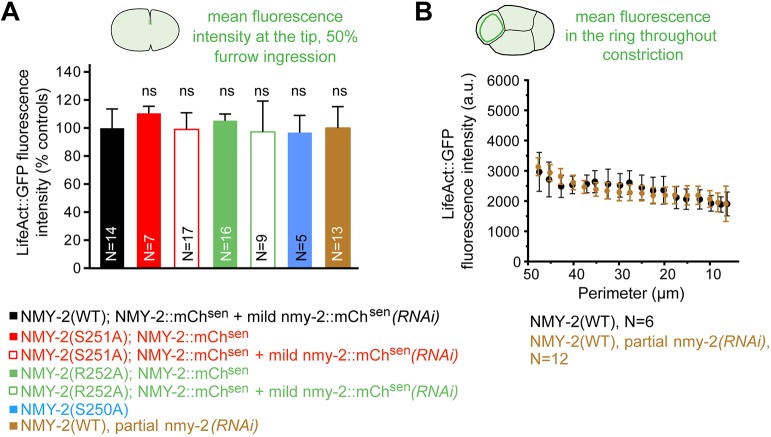
**Impairment of non-muscle myosin II motor activity does not affect F-actin levels in the constricting contractile ring.** (A) LifeAct::GFP levels (mean±95% CI) in the contractile ring at 50% furrow ingression in embryos with indicated genotypes and RNAi treatments (measured region is indicated in the schematic on top). Values were normalized to corresponding controls, which are NMY-2(WT);NMY-2::mCh^sen^ and NMY-2(WT). (B) Quantification of LifeAct::GFP levels (mean±95% CI) during ring constriction in the ABa cell of four-cell embryos, where the ring can be observed end-on. N is the number of embryos analyzed. Statistical significance was determined using one-way ANOVA followed by Bonferroni's multiple comparison test; ns, not significant (*P*>0.05).

We also examined whether myosin modulated actin levels in the contractile ring. We found that the concentration of LifeAct::GFP in the constricting ring at 50% ingression was unaltered in all conditions tested: one-cell embryos expressing NMY-2(S250A), embryos expressing NMY-2(S251A) or NMY-2(R252A) in the presence of normal or decreased levels of NMY-2::mCherry^sen^, and wild-type NMY-2-expressing embryos partially depleted of NMY-2 (Fig. 6A). In addition, as in control embryos, the concentration of LifeAct::GFP in the contractile ring remained constant throughout constriction after partial NMY-2 depletion (Fig. 6B). We conclude that neither lowering myosin levels nor impairing myosin motor activity affects actin levels in the constricting ring.

### Myosin motor activity is required for F-actin alignment at the division plane, compaction of the equatorial actin band and deformation of the equator

**Fig. 7.**
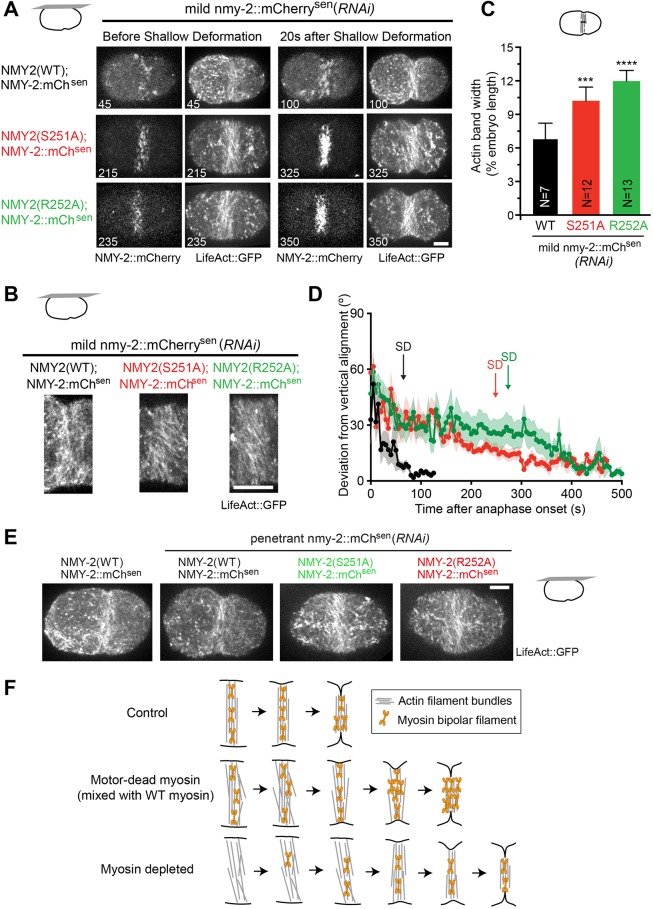
**Equatorial accumulation of motor-competent myosin is rate limiting for furrow initiation.** (A) Frames of time-lapse movies of one-cell embryos of the indicated genotypes co-expressing LifeAct::GFP and NMY-2::mCherry^sen^ after mild depletion of NMY-2::mCherry^sen^. Numbers on frames indicate time after anaphase onset. (B) Higher magnification of the cortical equatorial actin band in embryos of the indicated genotypes. (C) Width of equatorial actin band (mean±95% CI, normalized to embryo length) at shallow deformation for embryos of the indicated genotypes after mild depletion of NMY-2::mCherry^sen^. N is the number of analyzed embryos. (D) Deviation from vertical alignment of F-actin bundles (mean±s.e.m.) measured between anaphase onset and back-to-back membrane configuration in embryos of the indicated genotypes after mild depletion of NMY-2::mCherry^sen^. Average onset of equatorial shallow deformation (SD) is indicated. (E) Frames showing the equatorial actin band in embryos of the indicated genotypes after penetrant depletion of NMY-2::mCherry^sen^. (F) Summary of results. Statistical significance in C was determined using one-way ANOVA followed by Bonferroni's multiple comparison test; ****P≤0.0001; ***P≤0.001. Scale bars: 10 µm.

To better understand how decreasing NMY-2::mCherry^sen^ levels in embryos expressing myosin motor-dead mutants impacts contractile ring formation and furrow initiation, we examined F-actin bundle behavior and actomyosin recruitment to the cell equator (Fig. 7A,B, Movie 4). After mild depletion of NMY-2::mCherry^sen^ in control embryos, formation of an equatorial band of actin (LifeAct::GFP) and myosin (NMY-2::mCherry^sen^) was promptly followed by equatorial deformation. Analysis of F-actin bundle orientation at the equator revealed fast bundle alignment perpendicular to the anterior-posterior embryo axis with maximum bundle alignment occurring shortly after shallow deformation (Fig. 7D). In *nmy-2(S251A)* or *nmy-2(R252A)* embryos mildly depleted of NMY-2::mCherry^sen^, equatorial accumulation of actin occurred at a similar time to that in controls but deformation of the cell equator was substantially delayed. Myosin recruitment to the cell equator was slower in myosin mutants than in controls, and myosin levels increased beyond control levels during furrow initiation (Fig. 7A, Movie 4). Some F-actin bundles at the cell equator were slanted relative to the division plane (Fig. 7A,B), and the width of the actin equatorial band was broader than in controls (Fig. 7C). Analysis of F-actin bundle deviation from vertical alignment revealed that bundle alignment was slow and continued during furrow initiation (Fig. 7D). In *nmy-2(S251A)* or *nmy-2(R252A)* embryos depleted of NMY-2::mCherry^sen^ that failed to initiate furrowing, most F-actin bundles were slanted (Fig. 7E). Partial depletion of NMY-2 in otherwise wild-type embryos led to the formation of a broad equatorial actin band with some slanted bundles, but enough myosin accumulated to allow for bundle alignment and furrowing (Movie 5, Fig. S3E-G). Once equatorial myosin reached a level that corresponded to 13.9±0.1% of that of controls (Fig. S3D), equatorial deformation ensued.

Together, these results indicate that motor-dead myosin does not support furrowing and that a threshold of motor-competent myosin needs to be reached for furrow initiation. We conclude that myosin motor activity is required for equatorial band compaction, actin filament bundle orientation and equatorial deformation (Fig. 7F).

## DISCUSSION

### Myosin motor activity is required for cytokinesis

We combined *in vivo* and *in vitro* characterization of two motor-dead and two partially motor-impaired myosins to show that myosin motor activity is essential for cytokinesis in *C. elegans* embryos. Our data reveal that motor-dead NMY-2(S251A) and NMY-2(R252A), which bind but do not translocate F-actin, fail to support cytokinesis in the early embryo. *nmy-2(S251A)* or *nmy-2(R252A)* embryos completed cytokinesis in the presence of transgene-encoded wild-type NMY-2 but did so with slightly slower kinetics than controls. This indicates that motor-dead NMY-2 hinders the activity of wild-type NMY-2, perhaps through the formation of heterotypic filaments, like those made up of different non-muscle myosin II isoforms or different myosin classes (Beach et al., 2014; Billington et al., 2015; Shutova et al., 2014). Indeed, co-polymerization of different non-muscle myosin II isoforms was shown to result in the formation of filaments with intermediate motile properties (Melli et al., 2018). The importance of myosin motor activity is reinforced by the observation that embryos whose only source of non-muscle myosin is partially motor-impaired NMY-2(S250A) or NMY-2(R718C) were delayed in cytokinesis. In addition, *nmy-2(S250A)* embryos were more sensitive to a reduction in overall myosin levels than wild-type embryos, indicating that in the presence of motor-impaired myosin more myosin molecules are needed to complete cytokinesis.

Our results imply that the ability of myosin to crosslink F-actin is not sufficient for cytokinesis and therefore contrast with results in COS-7 cells and mouse cardiomyocytes, where myosin was proposed to exert tension in a motor-independent manner during cytokinesis (Ma et al., 2012). We note that of the three myosin mutants presumed to be motor-dead in that report, NMIIA(N93K) was recently shown not to be motor-dead (Heissler et al., 2018), and our *in vivo* characterization of NMY-2(R718C) and UNC-54(R710C), which are equivalent to NMIIB(R709C), strongly argues that this myosin mutant is also not motor-dead. This is in agreement with the characterization of the corresponding mutation in mammalian NMIIA, R702A, which was reported to translocate F-actin at half the velocity of wild-type NMIIA (Hu et al., 2002). The third myosin mutant analyzed by Ma et al. is NMIIB(R234A), which is equivalent to NMY-2(R252A). This mutant was recently proposed to be a bona fide motor-dead mutant in mammalian myosins (Heissler et al., 2018). NMIIB(R234A) was able to ameliorate cytokinesis defects in COS-7 cells depleted of NMIIB, while we show that NMY-2(R252A) does not support cytokinesis in the *C. elegans* embryo. The *Dictyostelium discoideum* mutant MYS2(R238A), which corresponds to NMY-2(R252A) and NMIIB(R234A), caused very slow cell growth when cultured in suspension, but supported apparently normal cytokinesis when cells grew adhered to a surface (Shimada et al., 1997). This indicates that cytokinesis operates differently when cells divide while adhering to a surface. Indeed, some adherent cultured cells can divide with a compromised contractile ring in an adhesion-dependent manner by migrating away in opposite directions during cell division (Dix et al., 2018; Kanada et al., 2005; Nagasaki et al., 2009; O'Connell et al., 1999).

### Myosin motor activity is required during furrow ingression

We observed that in embryos co-expressing motor-dead NMY-2(S251A) or NMY-2(R252A) with low levels of transgene-encoded wild-type myosin (i.e. after mild RNAi of NMY-2::mCherry^sen^), the rate of contractile ring constriction slowed substantially. These embryos were also delayed in contractile ring assembly and furrow initiation, and presented reduced F-actin bundle alignment and increased actin band width at the cell equator before furrow ingression. Therefore, it is possible that defects in F-actin architecture contribute to the slowdown in ring constriction in these mutants. However, embryos solely expressing partially motor-impaired NMY-2(S250A) or NMY-2(R718C), which bind F-actin similarly to wild-type NMY-2, assembled contractile rings with normal timing and appearance (Movie 3), yet were still delayed in furrow initiation and had a slower rate of ring constriction. Thus, ring constriction slowdown in *nmy-2(S250A)* and *nmy-2(R718C)* embryos is unlikely to be a consequence of improperly assembled contractile rings. This indicates that myosin motor activity continues to be required after the contractile ring is formed and that motor activity sets the contraction speed of the F-actin network of the ring. Our results therefore argue against the hypothesis that contractile stress induced by passive F-actin crosslinking combined with F-actin treadmilling is sufficient to drive ring constriction independently of myosin motor activity (Mendes Pinto et al., 2012; Oelz et al., 2015). Our data are in agreement with that obtained for contractile rings isolated from fission yeast expressing a myo2 mutant (myo2-E1-Sup1, carrying G345R, Q640H and F641I mutations) that binds tightly to actin but does not translocate F-actin *in vitro* (Palani et al., 2017). Contrary to controls, isolated mutant rings did not constrict in the presence of ATP. Interestingly, ∼65% of *S. pombe* cells expressing myo2-E1-Sup1 were able to complete cytokinesis because of the compensating activity of Myp2 (another myosin II isoform) and perhaps Myo51 (a myosin V), which are both normally dispensable (Laplante et al., 2015; Palani et al., 2017).

It is possible that myosin motor activity contributes to contractile ring constriction by determining F-actin turnover, as proposed previously (Guha et al., 2005; Mendes Pinto et al., 2012; Murthy and Wadsworth, 2005; Wilson et al., 2010). The concentration of actin in the contractile ring remains constant throughout constriction, which indicates that there is net depolymerization of F-actin as the ring becomes smaller (Carvalho et al., 2009). We observe that the rate of contractile ring constriction slows in myosin motor mutants, yet the concentration of actin in the ring at 50% of ingression is the same as in controls. This indicates that rings that constrict more slowly due to impaired myosin motor activity experience proportionally slower net depolymerization of F-actin such that the actin concentration in the ring remains the same as in controls.

Myosin has been implicated in disassembling F-actin networks (Backouche et al., 2006; Haviv et al., 2008; Medeiros et al., 2006; Reymann et al., 2012; Wilson et al., 2010), likely through F-actin buckling. F-actin buckling has been proposed to be caused by myosin filaments with slightly different velocities (Lenz et al., 2012), and F-actin buckles have a higher curvature and should therefore be more prone to severing (Murrell and Gardel, 2012; Schramm et al., 2017; Vogel et al., 2013). Our analysis of actin levels in the constricting ring in myosin motor mutants is consistent with the idea that myosin motor activity disassembles F-actin. Net depolymerization of F-actin could directly drive ring constriction or it could keep ring structure optimized for myosin-driven contractility.

### Equatorial accumulation of motor-competent myosin is rate limiting for furrow initiation

We show that cytokinesis is successful in embryos co-expressing equal amounts of wild-type and motor-dead myosin, and that similar amounts of NMY-2::mCherry^sen^ are recruited to the equator in *nmy-2(WT)*, *nmy-2(S251A)* and *nmy-2(R252A)* embryos (Movie 2). By contrast, when motor-dead myosin is present in excess of wild-type myosin, i.e. after mild depletion of NMY-2::mCherry^sen^ in *nmy-2(S251A)* or *nmy-2(R252A)* embryos, we observe enhanced recruitment of NMY-2::mCherry^sen^ to the cell equator (Fig. 7A, Movie 4). We speculate that the excess of motor-dead myosin may favor the formation of homotypic motor-dead filaments that hinder contractility. In this scenario, more wild-type myosin would need to be recruited to the equator to achieve sufficient contractility for furrow initiation. Our results also reveal that when NMY-2 is substantially depleted by RNAi in wild-type animals, equatorial deformation occurs when myosin levels at the equator have reached ∼14% of control levels (Movie 5). This is consistent with the idea that when total myosin levels are low, less equatorial myosin is required for furrow initiation because there is also less cortical tension that resists furrowing (Silva et al., 2016). Overall, our results support the idea that a threshold level of myosin-based contractility is required to initiate furrowing. We propose that the accumulation of motor-competent myosin at the equatorial region is rate limiting for furrow initiation and that proper F-actin alignment at the division plane, compaction of the equatorial actin band and deformation of the equator are dependent on myosin motor activity.

How myosin is recruited to the cell equator is still debated and may involve a diffusion-retention mechanism dependent on local RhoA activation (Bement et al., 2005; Kimura et al., 1996; Uehara et al., 2010), myosin mechanosensitivity and catch-bonding behavior (Capitanio et al., 2012; Guo and Guilford, 2006; Vernerey and Akalp, 2016), and/or equator-directed cortical flows (Bray and White, 1988; Khaliullin et al., 2018; Reymann et al., 2016). If equator-directed flows contribute to the accumulation of excess NMY-2::mCherry^sen^ in the ring when motor-dead myosin is present, then other cortical components, such as anillin and septins, may also accumulate in excess. It will also be interesting to address how myosin motor mutants affect cortical actin flows, which have been described to drive alignment and compaction of F-actin in the ring and to be myosin dependent (Reymann et al., 2016).

### Motor activity of major muscle myosin is dispensable for F-actin organization in adult muscle

We used motility assays to show that our myosin motor mutants were defective in translocating F-actin *in vitro*. However, this does not necessarily reflect the situation *in vivo*, where a variety of actin regulators co-exist. We used muscle contraction as an *in vivo* readout of motor-impairment in myosin II mutants. Animals expressing UNC-54(S240A) or UNC-54(R241A), which are equivalent to NMY-2(S251A) and NMY-2(R252A), respectively, displayed a dramatic reduction in movement (with residual movement depending on the other muscle myosin, MYO-3) and were unable to lay eggs. As both of these behaviors depend on the contraction of muscle sarcomeres, these results strongly suggest that the mutations result in motor-dead muscle myosin. Interestingly, and in contrast to UNC-54 depletions, F-actin organization in adult muscle was not overtly affected by motor-dead UNC-54. This is in agreement with results from flight muscles in *Drosophila melanogaster* that suggested that muscle myosin motor activity is largely dispensable for the high-order organization of F-actin during sarcomere maturation (Loison et al., 2018). The movement of animals expressing UNC-54(S239A), which is equivalent to NMY-2(S250A), was reduced but not abolished, in agreement with the prediction that this mutation produces partially motor-impaired myosin.

Overall, our data and previous studies of other myosin II proteins suggest that mutating specific highly conserved residues in the switch I loop of the ATP binding site consistently produces motor-dead and partially motor-impaired myosin in different myosin II families. Therefore, these mutations should be useful to study the role of myosin II motor activity in a variety of tissues.

## MATERIALS AND METHODS

### *C. elegans* strains

Strains used in this study and their genotypes are listed in Table S1 and were maintained on nematode growth medium (NGM) plates seeded with OP50 *E. coli* at 20°C using standard techniques (Stiernagle, 2006). Re-encoded nmy-2 transgene fused to mCherry in strain GCP22 was generated by Mos1-mediated single-copy transgene insertion (MosSCI) onto chromosome II, tTi5605 (Frøkjaer-Jensen et al., 2008; Frøkjær-Jensen et al., 2012). The nmy-2 promoter region (5.2 kb), ORF and 3′UTR (1.3 kb) were cloned as overlapping fragments from genomic DNA. A region of ∼400 bp in exon 12 was re-encoded so that transgenic nmy-2, and not endogenous nmy-2, could be specifically depleted by RNAi. mCherry was cloned from pDC122 and all fragments were assembled with pCFJ151 backbone through Gibson assembly (Gibson et al., 2009). Single-copy transgene insertions were generated by injecting a mixture of target plasmid pAC71, plasmid with transposase pCFJ601 and plasmids carrying the selection markers pCFJ90, pCFJ104 and pGH8 into the strain EG6429, as described previously (Frøkjaer-Jensen et al., 2008; Frøkjær-Jensen et al., 2012). Transgene integrations were confirmed by PCR of regions spanning each side of the insertion, by sequencing of the entire genomic DNA locus and by fluorescence microscopy.

To generate point mutants in *nmy-2* and *unc-54*, the endogenous loci of *nmy-2* and *unc-54* were modified using CRISPR/Cas9-mediated genome editing. Two or three single guide RNAs (sgRNAs) were cloned into the pDD162 vector (Dickinson et al., 2013) and injected together with a single-stranded repair template carrying the modified sequence of interest flanked by 35-50 bp homology regions (IDT ultramer) into gonads of young adult N2 hermaphrodites. All sgRNAs and repair templates used are listed in Table S2. To facilitate the identification of successfully injected animals, injection mixes contained a sgRNA and a repair template to generate the R92C mutation in the *dpy-10* gene, which causes a dominant roller phenotype (Arribere et al., 2014; Levy et al., 1993). All point mutations were verified by DNA sequencing. To remove potential off-target mutations, mutants were subjected to six rounds of outcrossing with N2 animals. *nmy-2(S251A)* and *nmy-2(R252A)* homozygous animals were sterile. To propagate these animals, two approaches were used. In the first approach, *nmy-2(S251A)* and *nmy-2(R252A)* animals were balanced with hT2 (from strain JK2739) to generate strains GCP629 and GCP513, respectively. Heterozygous mutant animals were fertile and easily identifiable, and homozygous animals were sterile (their gonads are shown in Fig. 2A). In the second approach, we were able to keep the *nmy-2(S251A)* and *nmy-2(R252A)* animals in homozygosity after crossing the strains GCP629 and GCP513 with GCP22 to generate the strains GCP618 and GCP592, respectively, which contained mutated endogenous *nmy-2* on chromosome I and transgenic *nmy-2* (re-encoded for RNAi resistance and fused to mCherry but otherwise wild type) on chromosome II. The expression of transgene-encoded NMY-2::mCherry kept *nmy-2(S251A)* and *nmy-2(R252A)* animals fertile and led to the production of viable embryos.

### RNA interference

RNAi was performed by feeding hermaphrodites with bacteria that express dsRNA. DNA fragments of target genes were cloned into the L4440 vector, which was transformed into HT115 *E. coli*. Primers in Table S3 were used to amplify *nmy-2* fragments from N2 cDNA (nmy-2_RNA#1) or from the synthesized re-encoded region (nmy-2_RNA#2). These fragments were cloned using the EcoRV restriction site in L4440. For RNAi of *myo-3* or *unc-54*, the corresponding L4440 vectors were obtained from the Ahringer library (Source Bioscience) and sequenced to confirm the gene target. Expression of dsRNAs in transformed HT115 was induced with 1 mM IPTG and the bacteria were fed to hermaphrodites as described below.

As protein levels in newly fertilized embryos gradually decrease with increasing duration of RNAi treatment of the mother (Kirkham et al., 2003; Velarde et al., 2007), different RNAi conditions were used to deplete NMY-2 depending on the desired level of depletion. RNAi treatment conditions were different depending on the use of nmy-2_RNA#1, nmy-2_RNA#2 or nmy-2_RNA#3:

In experiments where specific depletion of transgenic NMY-2::mCherry^sen^ was desired [labeled as nmy-2::mCherry^sen^(*RNAi*) in the figures], nmy-2_RNA#2 was used in GCP22, GCP618 or GCP592 L4 hermaphrodites at 20°C. Mild depletions (22-26 h of RNAi treatment) were used in Figs 4A,B,D,E, 6A, and 7A-D. Under these conditions, most embryos were able to complete cytokinesis. Penetrant depletions (36-38 h of RNAi treatment) were used in Figs 4B,C and 7E. In these conditions all one-cell embryos failed cytokinesis. More-penetrant depletions (38-48 h of RNAi treatment) were used in Fig. 3D. Under these conditions hermaphrodites presented non-compartmentalized gonads and stopped producing embryos, and embryos laid at earlier time points were not viable.

In experiments whose purpose was to deplete endogenous NMY-2 to an extent that did not preclude cytokinesis [labeled as nmy-2(*RNAi*) in the figures], nmy-2_RNA#1 was used in L4 hermaphrodites at 20°C (Fig. 6, Fig. S3). Mild depletions (26-28 h of RNAi treatment) were used in GCP21, GCP401 and GCP420 strains in Fig. 5F,H and Fig. S5H. Under these conditions most *nmy-2(S250A)* and all *nmy-2(R718C)* embryos were able to complete cytokinesis albeit more slowly than controls. Partial depletions (30-32 h of RNAi treatment) were used in strains GCP21 (Fig. 6B), GCP179 (Fig. S3A-C) and GCP22 (Fig. S3D-G). Under these conditions most embryos were able to complete cytokinesis albeit more slowly than controls. In Fig. S3D-G, both endogenous and transgenic NMY-2::mCherry were simultaneously depleted using nmy-2_RNA#3.

Depletion of MYO-3 was accomplished by incubating L1 hermaphrodites of strains GCP565 and GCP619 (Fig. 1D), GCP523 (Fig. S4A) and GCP524 (Fig. S5B) for 72 h at 20°C. Depletion of MYO-3, UNC-54 or simultaneous depletion of MYO-3 and UNC-54 in Fig. 1D,F,G, Figs S4A and S5B were performed by feeding L1 hermaphrodites of strain N2 for 72 h at 20°C. For double RNAi of *myo-3* and *unc-54*, bacteria expressing each of the constructs were mixed 1:1 before seeding RNAi plates (Fig. 1F).

### Live imaging

Gravid hermaphrodites were dissected and one-cell embryos were mounted in a drop of M9 (86 mM NaCl, 42 mM Na_2_HPO_4_, 22 mM KH_2_PO_4_ and 1 mM MgSO_4_) on 2% agarose pads overlaid with a coverslip. Live imaging of cytokinesis was performed at 20°C. Images were acquired on a spinning disk confocal system (Andor Revolution XD Confocal System; Andor Technology) with a confocal scanner unit (CSU-X1; Yokogawa Electric Corporation) mounted on an inverted microscope (Ti-E, Nikon) equipped with a 60×1.42 oil-immersion Plan-Apochromat objective, and solid-state lasers of 405 nm (60 mW), 488 nm (50 mW) and 561 nm (50 mW). For image acquisition, an electron multiplication back-thinned charge-coupled device camera (iXon; Andor Technology) was used. Acquisition parameters, shutters and focus were controlled using Andor iQ3 software. For central plane imaging in one-cell embryos, 6×1 μm *z* stacks were collected in the 488 nm and 561 nm channels every 10 s in embryos of GCP22, GCP592, GCP618, GCP21, GCP401, GCP420 and GCP179 strains. For cortical imaging in one-cell embryos, 7×0.5 μm *z* stacks were collected in the 488 nm and 561 nm channels every 5 s in embryos of GCP22, GCP592 and GCP618 strains. For imaging GCP401 and GCP420 embryos, the same protocol was applied using the 488-nm channel.

### Embryonic viability and egg laying assay

In Figs S4E and S5F, L4 hermaphrodites of strains GCP21, GCP401 or GCP420 were placed on NGM plates and singled out onto fresh plates after 40 h at 20°C. Adult gravid hermaphrodites were allowed to lay eggs for 8 h. In Fig. 3D, GCP22, GCP618 or GCP592 L4 hermaphrodites were placed on plates with bacteria expressing nmy-2_RNA#2 for 40 h at 20°C. Adult hermaphrodites were singled out onto fresh RNAi plates and left to lay eggs for 8 h. In all cases, animals were removed after the egg laying interval and embryos were left to hatch at 20°C for 24 h before counting. The number of hatched and unhatched (dead) embryos was counted and the fraction of viable embryos calculated by dividing the number of hatched embryos by the total number of progeny. To measure egg-laying rates in Fig. 1E, Figs S4B and S5C, L4 hermaphrodites of strains N2, GCP523, GCP524, GCP565 or GCP619 were placed on NGM plates and singled out onto fresh plates after 40 h at 20°C. Adult hermaphrodites were allowed to lay eggs for 8 h. The egg laying rate was calculated by dividing the total number of eggs on the plate by the number of hours during which eggs were laid.

### Worm protein extracts and immunoblotting

For protein sample preparation in Fig. 3C and Fig. S4D, 60 L4 hermaphrodites of strains N2, GCP22, GCP592 GCP618 or GCP401 were grown on NGM plates for 46 h at 20°C and pelleted at 750 ***g***. Animals were washed three times in M9 medium supplemented with 0.1% Triton X-100. Lysis was performed by resuspending the pellet in SDS-PAGE sample buffer [250 mM Tris (pH 6.8), 30% (v/v) glycerol, 8% (w/v) SDS, 200 mM DTT and 0.04% (w/v) bromophenol blue] with one-third the volume of quartz sand (Sigma). Tubes were subject to three 5 -min cycles of alternating boiling at 95°C and vortexing, after which the quartz sand was pelleted and the supernatant recovered. Protein samples were resolved by SDS-PAGE in a 7.5% gel and transferred to 0.2-μm nitrocellulose membranes (GE Healthcare). Membranes were blocked with 5% nonfat dry milk in TBST (20 mM Tris, 140 mM NaCl, and 0.1% Tween, pH 7.6) and probed at 4°C overnight with the following primary antibodies: anti-NMY-2 (1:10,000, home-made rabbit polyclonal antibody against residues 945-1368) and anti-α-tubulin (1:5000, mouse monoclonal DM1-α, T6199, Sigma). Membranes were washed three times with TBS-T and incubated for 1 h at room temperature with the following HRP-conjugated secondary antibodies: goat anti-rabbit (1:5000, 111-035-144, Jackson ImmunoResearch) and goat anti-mouse (1:5000, 115-035-003, Jackson ImmunoResearch). Membranes were washed three times with TBST and proteins were visualized by chemiluminescence using Pierce ECL Western Blotting Substrate (Thermo Fisher Scientific) and a ChemiDoc XRS+ System with Image Lab Software (Bio-Rad).

### Protein sequence alignments

Myosin protein sequences for alignments shown in Fig. 1B,C, Figs S1 and S5A were obtained from the Uniprot database with the following accession numbers: P08799|MYS2_DICDI; G5EBY3|G5EBY3_CAEEL; Q20641|Q20641_CAEELQ99323|MYSN_DROME; F1QC64|F1QC64_DANRE; F8W3L6|F8W3L6_DANRE; F1R3G4|F1R3G4_DANRE; 93522|O93522_XENLA; Q04834|Q04834_XENLA; Q8VDD5|MYH9_MOUSE; Q61879|MYH10_MOUSE; Q6URW6|MYH14_MOUSE; P35579|MYH9_HUMAN; P35580|MYH10_HUMAN; Q7Z406|MYH14_HUMAN; and P02566|MYO4_CAEEL. Sequences were aligned using Jalview software (Waterhouse et al., 2009) and the muscle algorithm with default parameters (Edgar, 2004).

### Protein expression and purification

Primers used for cloning of expression constructs are listed in Table S4, and plasmid information is provided in Table S5. DNA coding for wild-type NMY-2 S1 (amino acids 1-874), NMY-2 HMM (amino acids 1-1364), full-length MLC-4, full-length MLC-5 and full-length LET-502 was amplified from *C. elegans* cDNA and cloned into the pACEbac1 expression vector using Gibson assembly (Gibson et al., 2009). NMY-2 constructs were tagged N-terminally with 6×His followed by a flexible linker (GlySerGlySerGly). LET-502(1-469), MLC-4 and MLC-5 were tagged N-terminally with StrepTagII followed by a flexible linker (GlySerGlySerGly). To obtain the vectors for expression of NMY-2 point mutants (S250A, S251A and R252A), the pACEbac1 vector containing wild-type *nmy-2* cDNA was used as a template for site-directed mutagenesis. To obtain the pACEbac1 vector for expression of LET-502(1-469), the pACEbac1 containing full-length *let-502* cDNA was amplified using back-to-back primers to delete the coding sequence downstream of amino acid 469. The resulting vector was re-ligated in a single reaction combining PNK kinase for PCR product phosphorylation (NEB), DpnI for template removal by digestion (Thermo-Fisher Scientific) and DNA T4 ligase (NEB). Plasmids were transformed in DH5-alpha or TOP10 competent bacteria and all constructs were verified by Sanger DNA sequencing.

Bacmid recombination was performed in DH10EMBacY bacteria (Geneva Biotech) and Sf21 cells were transfected with each of the bacmids using X-tremeGene HP DNA Transfection Reagent (Roche). Virus production was performed as previously described (Bieniossek et al., 2008). For large-scale purification of NMY-2 S1 fragments or NMY-2 HMM fragments bound to MLC-4 and MLC-5 (referred to as NMY-2_S1_ and NMY-2_HMM_, respectively), corresponding baculoviruses were used to co-infect 500 ml cultures of Sf21 cells (0.8×10^6^ cells/ml, SFM4 medium; Hyclone). Cells were harvested by centrifugation at 3000 ***g*** for 5 min. Pellets were resuspended in lysis buffer [15 mM MOPS, 300 mM NaCl, 15 mM MgCl_2_, 0.1% Tween 20, 1 mM EDTA, 3 mM NaN_3_, 1 mM DTT (pH 7.3)] supplemented with EDTA-free Complete Protease Inhibitor Cocktail (Roche), sonicated and incubated for 20 min with 1 mM ATP to detach myosin from actin. Lysates were cleared by centrifugation at 34,000 ***g*** for 40 min. NMY-2_S1_ was purified by batch affinity chromatography using Strep-Tactin Sepharose (IBA). Beads were washed with wash buffer [10 mM MOPS, 500 mM NaCl, 5 mM MgCl_2_, 0.1% Tween 20, 1 mM EDTA, 3 mM NaN_3_, 1 mM DTT (pH 7.3)] supplemented with 1 mM ATP in the first wash and eluted on a gravity column with elution buffer [10 mM MOPS, 500 mM NaCl, 2.5 mM desthiobiotin, 3 mM NaN_3_, 1 mM DTT (pH 7.3)]. NMY-2_S1_ was further purified by size-exclusion chromatography using a Superose 6 increase 10/300 column (GE HealthCare) pre-equilibrated with 10 mM MOPS, 500 mM NaCl and 1 mM EDTA (pH 7.3). Fractions containing NMY-2_S1_ were pooled and concentrated in 50 MWCO Amicon Ultra-15 Centrifugal Filter Units (Merck-Millipore). NMY-2_HMM_ was purified by a tandem Strep-Tactin-6× His-tag affinity chromatography approach. The purification in Strep-Tactin Sepharose was similar to that used for NMY-2_S1_, except that Tween-20 was excluded from the lysis and wash buffers, and 10 mM imidazole was added to the lysis buffer. Eluates from Strep-Tactin affinity chromatography were incubated with Ni-NTA beads (Thermo Fisher Scientific), washed with wash buffer 2 [10 mM MOPS, 500 mM NaCl, 25 mM imidazole, 3 mM NaN_3_, 1 mM DTT (pH 7.3)] and eluted in elution buffer 2 [10 mM MOPS, 500 mM NaCl, 250 mM imidazole, 3 mM NaN_3_, 1 mM DTT (pH 7.3)]. The eluate was concentrated using 50 MWCO Amicon Ultra-15 Centrifugal Filter Units (Merck-Millipore) and dialyzed overnight in low-salt buffer [10 mM MOPS, 25 mM KCl, 2 mM MgCl_2_, 1 mM EDTA (pH 7.3)]. After dialysis, the samples were centrifuged at 2000 ***g*** for 5 min and supernatants were retained. For both NMY-2_S1_ and NMY-2_HMM_, glycerol and DTT were added to a final concentration of 10% (vol/vol) and 1 mM, respectively. Aliquots were flash-frozen in liquid nitrogen and stored at −80°C.

For LET-502(1-469) expression, baculoviruses were used to infect large-scale Sf21 cultures and protein was extracted from cells following similar procedures as described above but using the same buffer for lysis and washes [50 mM Tris, 150 mM NaCl, 3 mM NaN_3_ 10% glycerol, 1 mM DTT (pH 7.3)] supplemented with EDTA-free Complete Protease Inhibitor Cocktail (Roche). LET-502(1-469) was purified by batch affinity chromatography using Strep-Tactin Sepharose (IBA). Beads were washed with lysis/wash buffers and eluted on a gravity column with elution buffer 3 [50 mM Tris, 150 mM NaCl, 3 mM NaN_3_ 10% glycerol, 2.5 mM desthiobiotin (pH 7.3)]. Eluates were concentrated in a 10 MWCO Amicon Ultra-15 Centrifugal Filter Units (Merck-Millipore) and dialyzed overnight in dialysis buffer 2 [50 mM Tris, 150 mM NaCl, 3 mM NaN_3_, 10% glycerol (pH 7.3)].

### F-actin co-sedimentation assay

G-actin was from lyophilized human platelets (APHL99, Cytoskeleton) or rabbit muscle. Rabbit muscle G-actin was extracted from muscle acetone powder (Sigma, M6890) as described previously (Sellers, 1998) and lyophilized by freeze-drying after adding 2 mg of sucrose per mg of G-actin. Lyophilized G-actin was resuspended in water at 10 mg/ml and diluted to 1 mg/ml in G-actin buffer with ATP [5 mM Tris, 0.2 mM CaCl_2_, 0.2 mM ATP (pH 8)]. Polymerization was induced by addition of 10× F-actin buffer [1× F-actin buffer: 5 mM Tris, 0.2 mM CaCl_2_, 50 mM KCl, 2 mM MgCl_2_, 1 mM ATP (pH 8)]. F-actin used in each round of co-sedimentation assays was from the same batch and source. Wild-type or mutant NMY-2_S1_ was dialyzed overnight in F-actin buffer without ATP and supplemented with 1 mM DTT. All samples were pre-cleared by centrifugation at 150,000 ***g*** for 1 h in an Optima XP centrifuge with a TLA-100 rotor (Beckman-Coulter) prior to the assay. For the co-sedimentation assay, an equal amount (∼2 µM) of NMY-2_S1_ was incubated with F-actin stock (final concentrations were 14.7 µM F-actin and 0.7 mM ATP) or a similar volume of F-actin buffer (negative control) for 30 min at room temperature. A similar approach was used for the estimation of the dissociation constant (Kd): 0.5 µM NMY-2_S1_ was incubated with different amounts of actin as indicated in the plots in Figs 2E and 5C. Samples were centrifuged at 150,000 ***g*** for 1.5 h at room temperature. Supernatants (SIs) were removed and SDS-PAGE sample buffer was added to 1× final concentration. Pellets (PI) were resuspended in 30 µl of water and mixed by pipetting every 2 min over a period of 10 min on ice. SDS-PAGE sample buffer was added to 1× final concentration. To determine the ability of NMY-2_S1_ to detach from F-actin, pellets prepared as above were resuspended in F-actin buffer supplemented with 50 mM ATP (without F-actin) over a period of 10 min on ice. After 30 min of incubation at room temperature, samples were centrifuged at 150,000 ***g*** for 1.5 h at room temperature. Supernatant and pellet fractions (SII, PII) were collected and prepared for loading in gels as described above. After addition of SDS-PAGE sample buffer, samples were incubated for 4 min at 95°C to denature proteins and run on 20-4% TGX gradient precast gels (Bio-Rad) in running buffer (25 mM Tris, 192 mM glycine, 0.1% SDS). Gels were stained with Blue Safe Coomassie (NZYtech) according to manufacturer's instructions (Figs 2C,F,H, 5A, Figs S2 and S5D). To verify the proteins bands corresponding to NMY-2 S1 fragments, immunoblotting with the mouse His-H8 antibody against the 6xHis tag (1:2500, 05-949 Merck Millipore) and a goat anti-mouse HRP-conjugated secondary antibody (1:5000, Jackson ImmunoResearch) was performed as described in the immunoblotting section.

### *In vitro* phosphorylation assay

*In vitro* phosphorylation of MLC-4 was performed according to Haldeman et al. (2014). NMY-2_HMM_ (20 µg) were mixed with 0.5 µM of LET-502(1-469) in phosphorylation buffer [20 mM MOPS (pH 7.3), 0.1 mM EGTA, 5 mM MgCl_2_, 50 mM NaCl, 1 mM CaCl_2_]. The phosphorylation reaction was initiated by adding 2 mM ATP to the mix. The mix was incubated for 1 h at 24°C. The samples were precipitated with three volumes of acetone at −20°C and centrifuged at 15,000 ***g*** for 5 min. Acetone was removed and protein pellets were resuspended in urea sample buffer [8 M urea, 33 mM Tris-glycine (pH 8.6), 0.17 mM EDTA, 10 mM DTT added immediately before use] to a final concentration of ∼3.3 µg/µl. The entire sample was loaded without prior heating and separated in 4-20% TGX gradient 10-well precast gels (Bio-Rad) with Tris-glycine running buffer. Gels were stained with Blue Safe Coomassie stain (NZYtech) according to the manufacturer's instructions (Fig. 2H).

### *In vitro* motility assay

*In vitro* motility assays were performed according to Sellers (1998). Actin was polymerized from lyophilized G-actin as described for co-sedimentation assays and a 2 µM stock was labeled by incubating F-actin overnight with rhodamine-phalloidin (1 unit of phalloidin for 100 µl of F-actin; Molecular Probes, Thermo Fisher Scientific) in labelling buffer [10 mM MOPS (pH 7.3), 0.1 mM EGTA, 3 mM NaN_3_]. Motility chambers consisted of a microscopy slide taped to a 18×18 cm No. 1.5H coverslip (Marienfeld, Germany) previously coated with 1% nitrocellulose (2% collodion solution for microscopy, diluted to 1% with amyl acetate; Sigma-Aldrich). Two pieces of double-sided tape (Tesa) were used to form a ∼7 mm channel inside the chamber. NMY-2_HMM_ at 1 µg/µl in a high-salt solution [20 mM MOPS (pH 7.3), 0.1 mM EGTA, 5 mM MgCl_2_, 500 mM NaCl] were flowed into the chamber. BSA solution [20 mM MOPS (pH 7.3), 0.1 mM EGTA, 5 mM MgCl_2_, 500 mM NaCl, 1% BSA] was flowed to block the sites of the coverslip that did not bind NMY-2_HMM_. Low-salt buffer [20 mM MOPS (pH 7.3), 0.1 mM EGTA, 5 mM MgCl_2_, 50 mM NaCl] was used to remove unbound BSA. To block dead myosin heads and induce phosphorylation of MLC-4, the chamber was incubated with a solution containing unlabeled actin, LET-502(1-469) and ATP [20 mM MOPS (pH 7.3), 0.1 mM EGTA, 5 mM MgCl_2_, 50 mM NaCl, 1 mM CaCl_2_, 0.5 µM LET-502 (1-469), 5 µM unlabeled actin, 1 mM ATP] for four minutes. After washing, labeled F-actin solution [20 nM rhodamine-phalloidin F-actin, 1 mM DTT, 20 mM MOPS (pH 7.3), 0.1 mM EGTA, 5 mM MgCl_2_, 50 mM NaCl] was flowed into the chamber. Unbound labeled F-actin was removed with low-salt buffer and assay buffer added [20 mM MOPS (pH 7.3), 0.1 mM EGTA, 5 mM MgCl_2_, 25 mM KCl, 50 mM DTT, 1 mM ATP and 0.7% methylcellulose solution]. Chambers were imaged at 21°C every 2 s for 300 s total, using the microscope setup described above and the perfect focus system (Nikon) to maintain focus overtime.

### F-actin and DNA labelling of body wall muscles and gonads

Phalloidin labelling of muscle actin shown in Fig. 1G and Fig. S4C was carried out in N2 adult animals depleted of UNC-54 or in adult hermaphrodites of strain GCP523, GCP565 and GCP619. Animals were collected and washed twice in M9 buffer, fixed with 4% paraformaldehyde (20% aqueous solution, Electron Microscopy Sciences), diluted in 1x cytoskeleton buffer (10 mM MES-KOH pH 6.1, 138 mM KCl, 3 mM MgCl_2_, 2 mM EGTA) containing 0.32 M sucrose (Cramer and Mitchison, 1993) for 15 min, permeabilized with acetone at −20°C for 5 min, washed with PBS containing 0.5% Triton X-100 and 30 mM glycine (PBS-TG) for 10 min, and stained with 1:40 Oregon Green-phalloidin (Molecular Probes, Thermo Fisher Scientific) in PBS-TG for 30 min. After three 10 min washes with PBS-TG, animals were mounted with ProLong Antifade containing DAPI (Molecular Probes, Thermo Fisher Scientific). Labelling of gonads shown in Fig. 3A was carried out as described above in animals of strain N2, GCP513 and GCP623, but only the DAPI staining of the gonads is shown.

In Fig. 4A, DNA labelling of gonads was carried out in living adult hermaphrodites of strain GCP22, GCP592 and GCP618 depleted of NMY-2::mCherry^sen^. Animals were transferred to a drop of water containing OP50 bacteria (1:10 dilution of a saturated culture) and 2 mg/ml Hoechst 33342 (Thermo Fisher Scientific) to label DNA and incubated for 1.5 h. After a recovery period of 30-60 min in NGM plates seeded with OP50, animals were anesthetized with levamisole (1 mg/ml, Sigma) and mounted on 2% agarose pads for imaging.

### Image analysis, quantification and statistics

All microscopy image processing and measurements were carried out using Fiji (ImageJ; National Institutes of Health) (Schindelin et al., 2012) and Matlab (MathWorks). *Z*-stacks taken on the cell cortex were projected using the maximum intensity projection tool. Images within each figure panel are scaled equally. The equatorial region of the central plane was selected to create the kymographs shown in Figs 4C,D, 5F, Fig. S3C, using the Make Montage tool. Selected regions of 4 s time lapse movies (presented in Movie 1 for wild type, S251A and R252A NMY-2_HMM_) were used to create 300 s time projections presented in Figs 2I and 5D by using the Temporal Color Code tool with the fire color scale.

Coomassie-stained SDS-PAGE gels were digitized in a GS-800 Calibrated Imaging Densitometer (Bio-Rad) and relative band intensity quantified in Image-Lab 5.2.1 (Bio-Rad). For each myosin mutant, the ability of NMY-2_S1_ to co-sediment with F-actin was quantified by dividing the intensity of the band corresponding to the NMY-2 S1 fragment in the pellet by the sum of intensities of the NMY-2 S1 fragment bands in the supernatant and pellet. Graph plotting, linear regressions and statistical analyses were performed with Prism 7 or 8 (GraphPad Prism Software). Dissociation constant (Kd) estimations in Figs 2E and 5C were performed by fitting a ‘one-site specific binding’ model using a least squares nonlinear regression (GraphPad Prism Software). Error bars represent the 95% CI of the mean, except in Figs 2E and 5C where they represent the s.d., and in Fig. 7D where they represent the s.e.m. Statistical significance tests were performed using a Student *t*-test or one-way ANOVA with Bonferroni correction for multiple comparisons as indicated in figure legends.

### Measurement of cytokinesis, ring assembly and furrow initiation time intervals, and ring constriction rate

Measurement of cytokinesis, ring assembly and furrow initiation time intervals, as well as ring constriction rate were performed in one-cell embryos of the following strains: GCP22, GCP592 and GCP618 (Fig. 4E); GCP21 and GCP401 (Fig. 5G-H); GCP21 and GCP420 (Fig. S5G-H); and GCP179 (Fig. S3B). This analysis only included embryos that completed ring constriction. Cytokinesis time was the interval between anaphase onset (myosin or Lifeact signal are cytoplasmic but absent from chromosomes, allowing the identification of the moment when the metaphase plate devoid of signal transitions into two separated DNA masses at anaphase) and the time when the contractile ring reached a diameter of ∼5 µm. Ring assembly time was the time interval between anaphase onset and the establishment of a shallow deformation in the equatorial region (time point when the equator shows first sign of deformation). Furrow initiation time corresponded to the time interval between the establishment of the shallow deformation and the time when the plasma membranes of the nascent daughter cells became juxtaposed to one another (back-to-back membrane configuration). During ring constriction, the distance between the two sides of the ring was measured in the z-plane where this distance was widest at each time point and plotted against time. Ring constriction rate was the slope of the linear region between ∼70% and 30% ingression. All intervals and ring constriction rate were determined based on imaging of the embryo central plane.

### Fluorescence intensity measurements

To quantify the levels of Lifeact::GFP at the tip of the cytokinetic furrow in one-cell embryos, the mean fluorescence intensity in a 10-pixel wide, 30-pixel long (1.8×5.3 µm) region drawn over the tip of the furrow at 50% ingression was determined and the mean camera background was subtracted (a 40×40 pixel region placed outside of the embryos; Fig. 6A). The average intensity at the furrow tip is presented as a percentage of the corresponding controls. In Fig. 6B, quantification of actin levels in the contractile ring of ABa cells was carried out in 4-cell embryos of strain GCP22 by measuring the mean LifeAct::GFP fluorescence intensity in a manually traced 0.7 µm line over the circumference of the ring. The mean fluorescence intensity in a circle drawn in the cytoplasmic region at each time point was subtracted. Before quantification, each time-lapse movie was corrected for fluorescence intensity decay using the ImageJ bleach correction tool and the simple ratio method. Data from multiple rings were pooled and plotted against ring perimeter. The mean of data points that fell in overlapping 5 µm intervals was calculated and plotted against the perimeter at the center of each interval.

In Fig. S3D, NMY2::mCherry^sen^ intensity was calculated by measuring the average fluorescence intensity of a 130×35 pixel region placed over the cortical equatorial band and the camera mean fluorescence background was subtracted (a 40×40 pixel region placed outside of the embryos).

### Analysis of band width and bundle alignment at the equatorial cortex

Equatorial actin band width in Fig. 7C was the length of a line traced across the band, perpendicularly to the division plane in cortical projections at shallow deformation. Values were normalized to embryo length along the anterior-posterior axis measured in the central plane, and averaged for each condition. Deviation from vertical alignment (°) over time in Fig. 7D was measured using the Directionality plugin for ImageJ. A region of 30×70 pixels corresponding to the furrow region was selected for each movie. The average directionality (angle α, in degrees), was calculated for every frame of each movie using the local gradient orientation method. Deviation from vertical alignment (in degrees) was calculated for each angle α, by subtracting the value of this angle from 90°. Absolute numbers were plotted.

### Liquid thrashing assay

Hermaphrodites of strains N2, GCP523, GCP524, GCP565 or GCP619 were synchronized at the L1 stage by alkaline bleach treatment of adult gravid hermaphrodites (0.8% bleach, 250 mM NaOH; Stiernagle, 2006) to extract embryos, which were left to hatch overnight in M9 medium. L1 hermaphrodites were plated and grown at 20°C. Young adult hermaphrodites were transferred to an M9 droplet and left to acclimatize for 2 min after which images were acquired at ∼40 fps average in a SMZ 745T stereoscope (Nikon) mounted with a QIClic CCD camera (QImaging) and controlled by Micro-Manager software (Open Imaging). Body bends swimming frequencies in Fig. 1D,F and Figs S4A and S5B were automatically quantified using the wrMTrck plug-in using standard parameters (Nussbaum-Krammer et al., 2015) in ImageJ. Image background was removed by subtracting the average intensity projection of the stack and animals were segmented using Otsu intensity thresholding.

## Supplementary Material

Supplementary information
