# Supplementary material for: Crosslinking activity of non-muscle myosin II is not sufficient for embryonic cytokinesis in C. elegans
Source: Development. 2019 Nov 12;146(21):dev179150. doi: 10.1242/dev.179150 (PMC6857588; doi:10.1242/dev.179150)
Supplement: Supplementary information [file develop-146-179150-s1.pdf]

Osorio et al, Figure S1

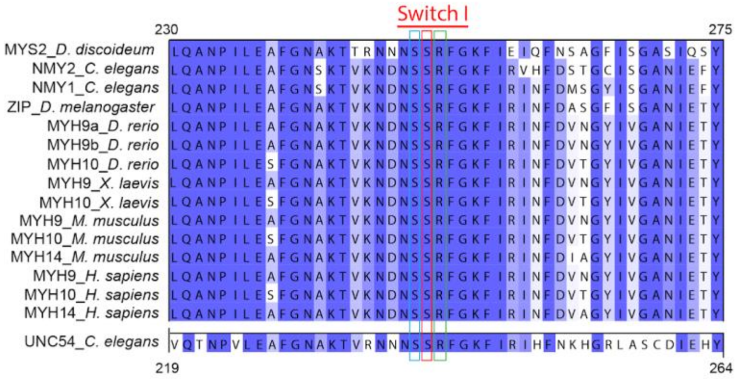

Figure S1. Conservation between non-muscle myosin IIs from different species

Alignment of non-muscle myosin IIs of several species along with *C. elegans* muscle myosin heavy chain B (UNC-54) showing conservation of the ATPase domain containing the switch I motif. Residues mutated in this study are boxed in cyan, red and green.

Development • Supplementary information

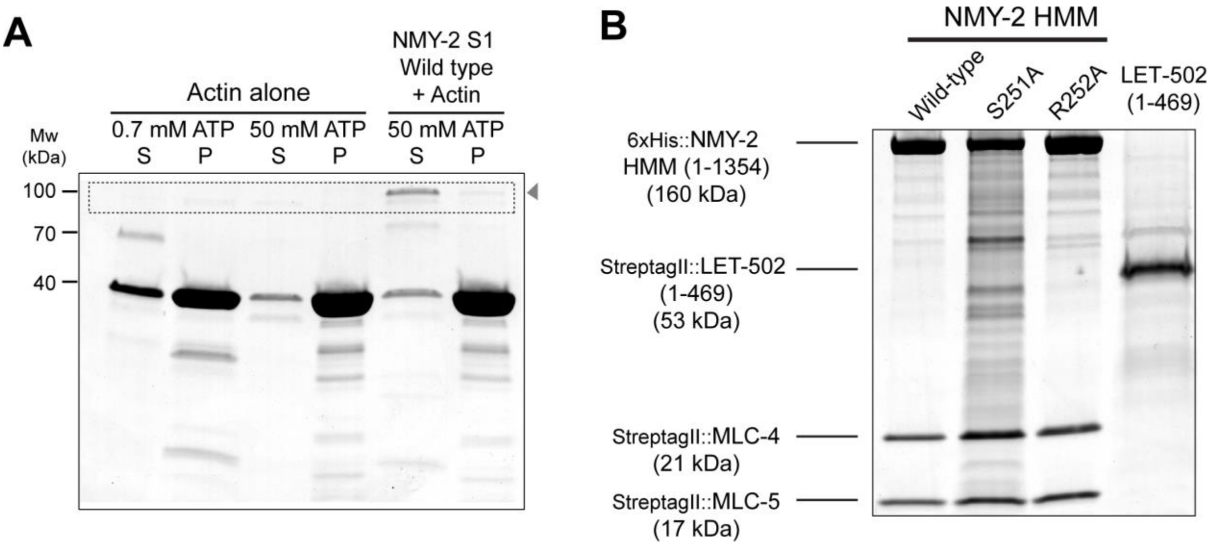

Figure S2. F-actin samples used in high-speed co-sedimentation assays and NMY-2$_{HMM}$ and LET-502(1-469) used for motility assays.

(A) Coomassie-stained SDS-PAGE gel of high-speed F-actin co-sedimentation in the absence of myosin showing supernatant and pellet fractions I in the presence of 0.7 mM ATP (lanes 1 and 2) and after resuspension with 50 mM ATP (lanes 3 and 4), or in the presence of wild-type NMY-2 and resuspended in 50 mM ATP. **(B)** Coomassie-stained SDS-PAGE gel of purified NMY-2(1-1354) HMM fragments in complex with MLC-4 and MLC-5 (lanes 1-3), and purified LET-502(1-469).

Development • Supplementary information

Osorio et al, Figure S3

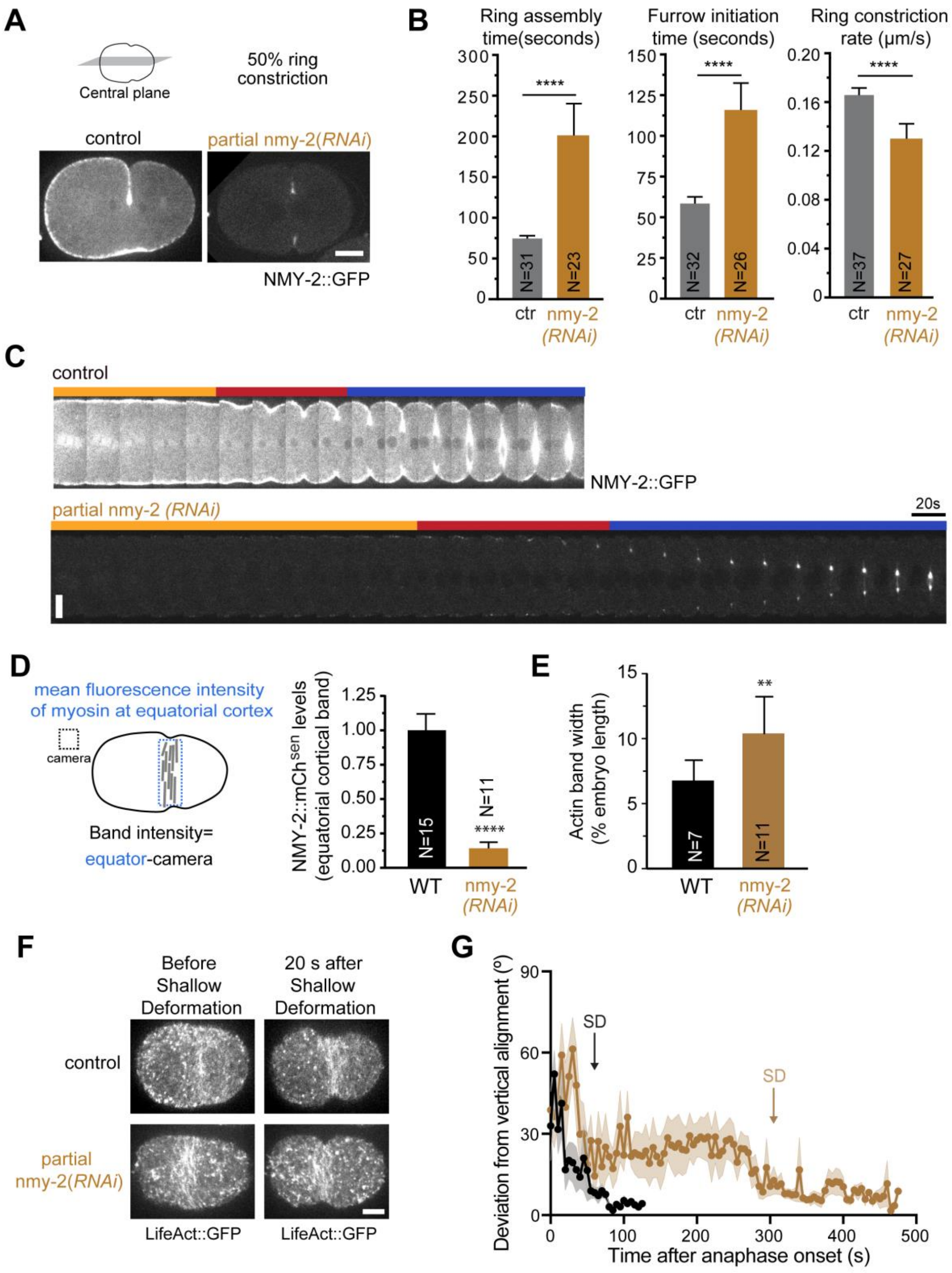

Development • Supplementary information

Figure S3. Partial depletion of NMY-2 in wild-type embryos allows cytokinesis to complete, albeit more slowly than in controls

(A) Central plane at 50 % furrow ingression of a control embryo and an embryo partially depleted of NMY-2 expressing NMY-2::GFP. **(B)** Ring assembly and furrow initiation time intervals and rate of ring constriction (mean±95% CI). **(C)** Kymographs of furrow region. Time zero corresponds to anaphase onset. Orange, red and blue bars indicate the intervals of ring assembly, furrow initiation and ring constriction, as depicted in Fig. 4D. (**D**) Quantification of mean NMY-2::mCherry^{sen} levels at the equatorial cortex, measured as indicated on the left. Note that *nmy-2(RNAi)* depletes both endogenous NMY-2 and transgene-encoded NMY-2::mCherry^{sen}. N is the number of embryos analyzed. (**E**) Equatorial actin band width normalized to embryo length at shallow deformation. **(F)** Stills of one-cell embryos expressing LifeAct::GFP before and after equatorial shallow deformation. **(G)** Deviation from vertical alignment of F-actin bundles (mean±SEM), measured between anaphase onset and back-to-back membrane configuration. Average onset of equatorial shallow deformation (SD) is indicated. Statistical significance was determined using the t-test; **** P≤0.0001, ** P≤0.01. Scale bars, 10 µm.

Development • Supplementary information

Osorio et al, Figure S4

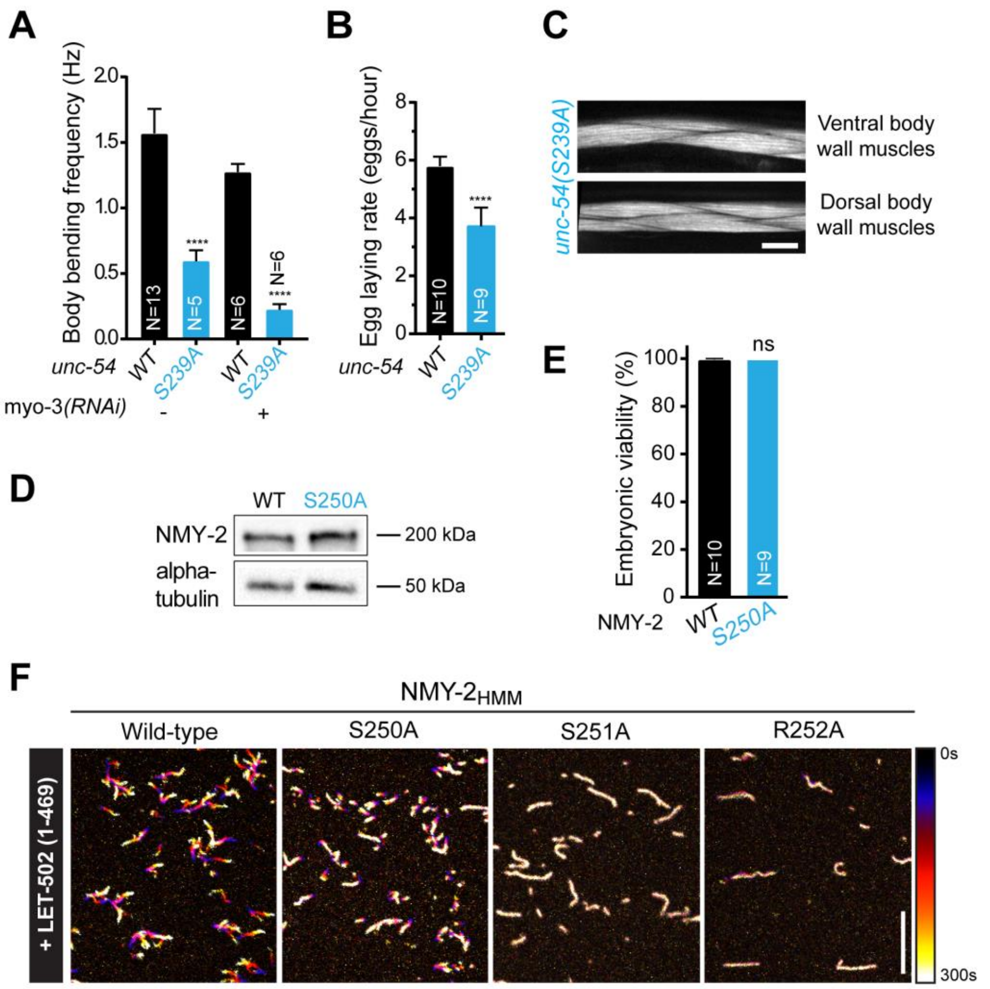

Figure S4. Additional characterization of NMY-2(S250A) and UNC-54(S239A) mutants

(A) Body bend frequency in liquid (mean±95% CI) in wild-type and *unc-54(S239A)* animals with and without depletion of MYO-3. **(B)** Egg laying rate (mean±95% CI) in wild-type and *unc-54(S239A)* animals. **(C)** Phalloidin staining of body wall muscles in *unc-54(S239A)* animals. **(D)** Immunoblot showing NMY-2 levels in wild-type and *nmy-2(S250A)* animals. α-tubulin is used as loading control. **(E)** Embryonic viability (mean±95% CI) in wild-type or *nmy-2(S250A)* animals. **(F)** Time projections of movies of F-actin sliding in the presence of wild-type, S250A, S251A or R252A NMY-2 HMMs after phosphorylation by LET-502(1-469). Color coding was used from black (0s) to white (300s). N is the number of animals analyzed in A and B and the number of animals whose progeny was analyzed in E. Statistical significance was determined using one-way ANOVA followed by Bonferroni's multiple comparison test; **** P≤0.0001, ns=not significant (P>0.05). Scale bars, 10 µm.

Osorio et al, Figure S5

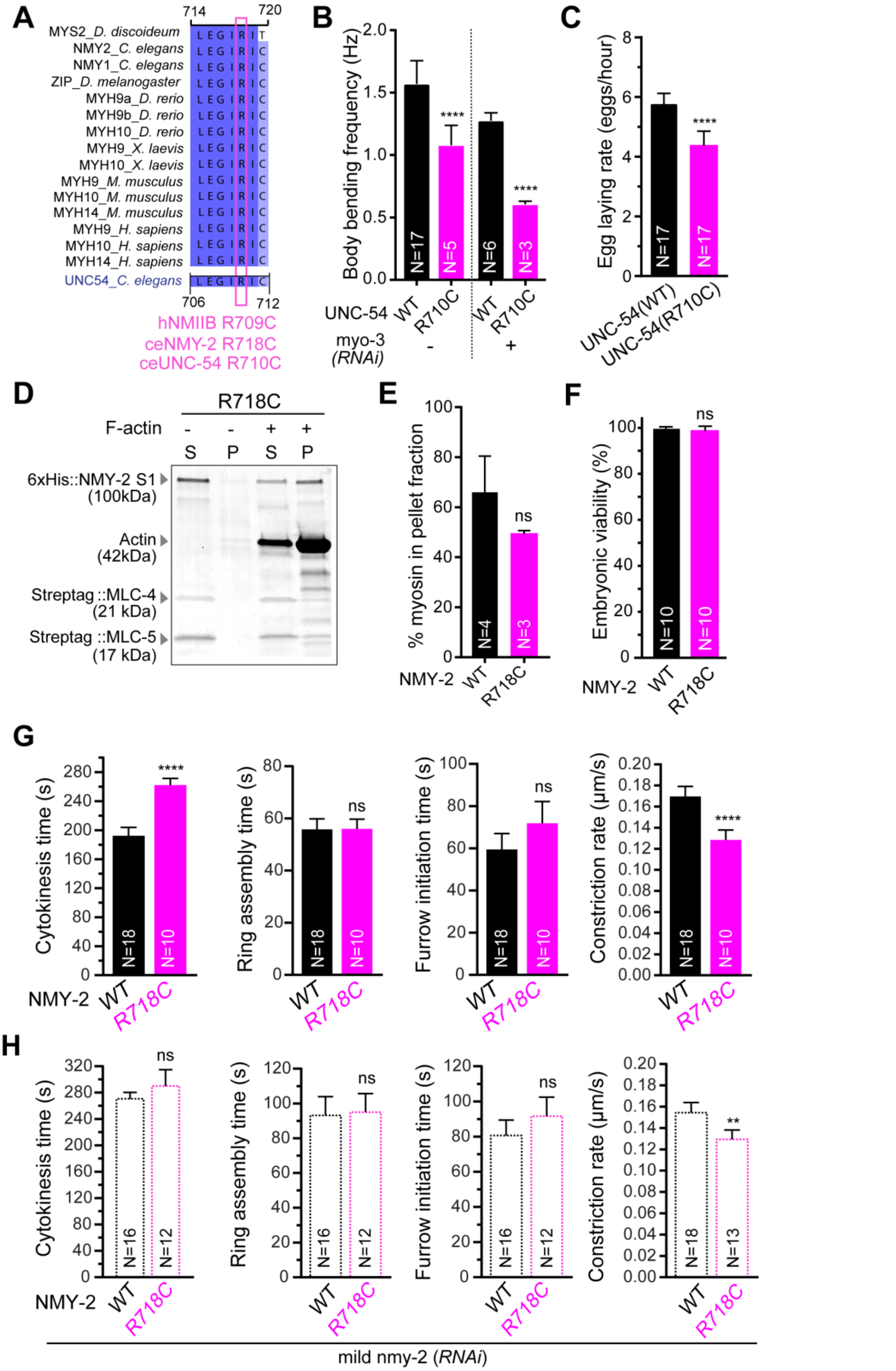

Development • Supplementary information

Figure S5. NMY-2(R718C), which is equivalent to human NMIIB(R709C), is not motor-dead

(A) Alignment of non-muscle myosin IIs of several species along with *C. elegans* muscle myosin heavy chain UNC-54 showing conservation of the SH1 helix region. **(B)** Body bend frequency in liquid (mean±95% CI) in wild-type and *unc-54(R710C)* animals with and without depletion of MYO-3. **(C)** Egg laying rate (mean±95% CI) in wild-type and *unc-54(R710C)* animals. **(D)** Coomassie-stained SDS-PAGE gel of high-speed F-actin co-sedimentation assays in which the NMY-2(R718C)$_{S1}$ was incubated with and without 14.7 µM of F-actin before ultracentrifugation. (S) indicates the supernatant and (P) the pellet fractions. **(E)** Percentage (mean±95% CI) of NMY-2$_{S1}$ present in the pellet, determined by measuring protein band intensities in Coomassie-stained SDS-PAGE gels as shown in *(D)*. **(F)** Embryonic viability (mean±95% CI) in wild-type or *nmy-2(R718C)* animals. **(G, H)** Cytokinesis, ring assembly and furrow initiation time intervals and rate of ring constriction (mean±95% CI) in wild-type and *nmy-2(R718C)* embryos with and without mild depletion of NMY-2. N is the number of analyzed animals in B and C, the number of animals whose progeny was analyzed in F, the number of independent experiments in E, and the number of analyzed embryos in G and H. Statistical significance was determined using one-way ANOVA followed by Bonferroni's multiple comparison test; **** P≤0.0001, ** P≤0.01, ns=not significant (P>0.05).

Development • Supplementary information

Movies

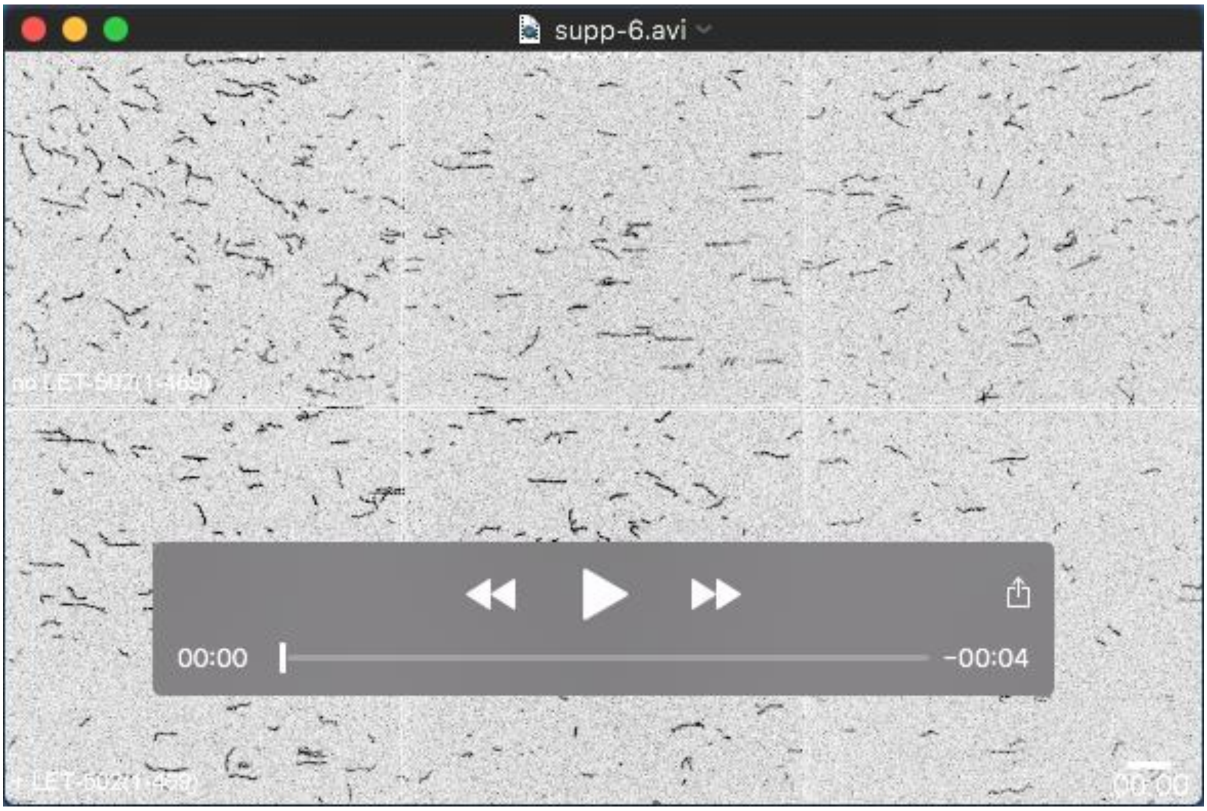

Movie 1. *In vitro* motility assay of wild-type or mutant NMY-2$_{HMM}$ phosphorylated by LET-502(1-469)

Time-lapse images of Rhodamine-phalloidin-labelled F-actin filaments in motility chambers coated with NMY-2(wild-type)$_{HMM}$, NMY-2(S251A)$_{HMM}$ or NMY-2(R252A)$_{HMM}$ in the absence (top row) or presence of LET-502(1-469). Images were taken every 4 seconds. Frame rate 20 fps, timestamps min:sec. Scale bar, 10 µm.

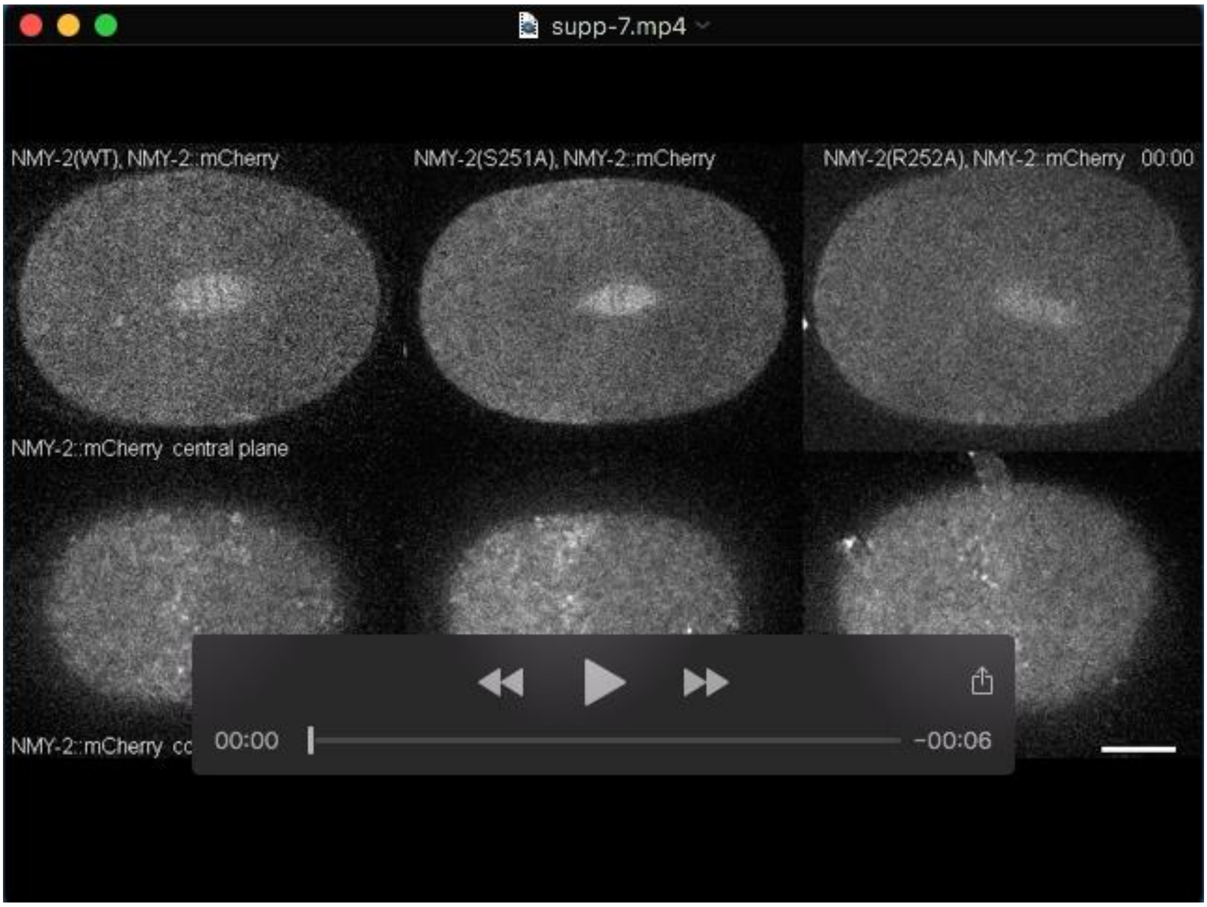

Movie 2. NMY-2::mCherry^{sen} distribution in embryos expressing wild-type or motor-dead myosin

Time-lapse images of the cell cortex (top row) and central plane (bottom row) in dividing one-cell embryos expressing NMY-2::mCherry^{sen} together with endogenous unlabelled NMY-2(wild-type), NMY-2(S251A) or NMY-2(R252A). Cortical images on the top row are maximum intensity projections of seven z-sections 0.5 µm apart taken every 5 seconds from anaphase onset (0:00). Frame rate 5 fps, timestamps min:sec. Scale bar, 10 µm.

Development • Supplementary information

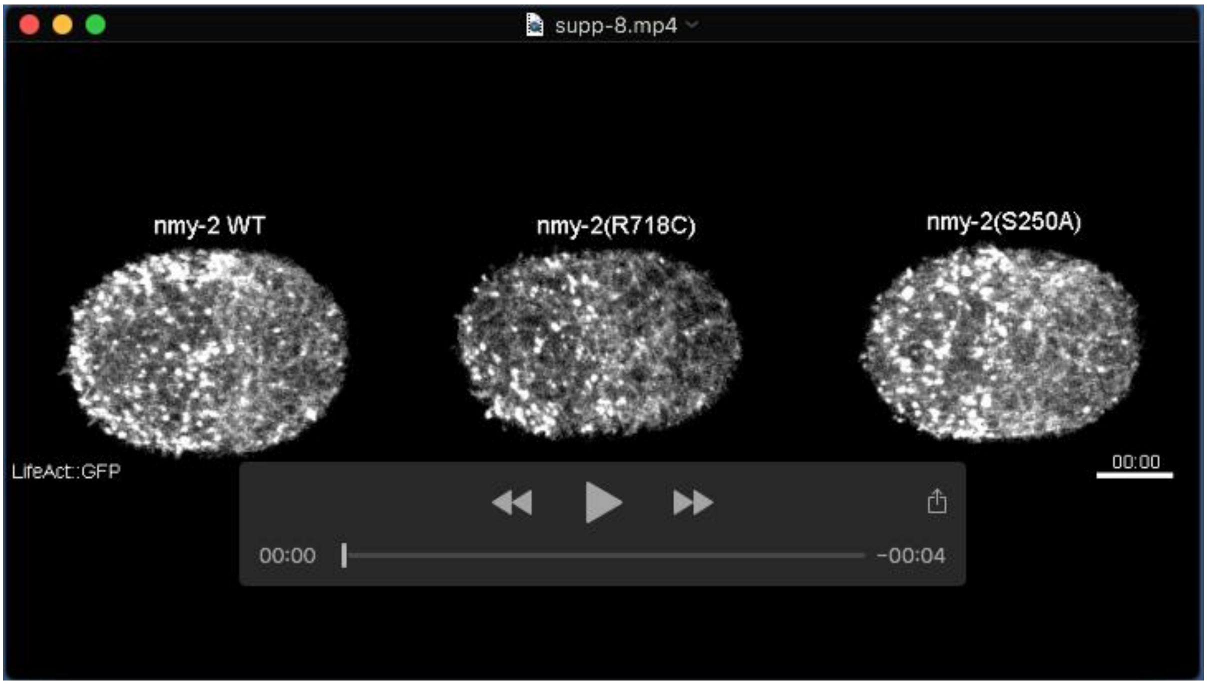

Movie 3. Cortical actin equatorial band in embryos expressing NMY-2(S250A) and NMY-2(R718C) is similar to that of controls

Time-lapse images of the cell cortex in dividing wild-type, *nmy-2(S250A)* and *nmy-2(R718C)* embryos one-cell embryos expressing LifeAct::GFP. Images are maximum intensity projections of seven z-sections 0.5 µm apart taken every 5 seconds from anaphase onset (0:00). Frame rate 5 fps, timestamps min:sec. Scale bar, 10 µm.

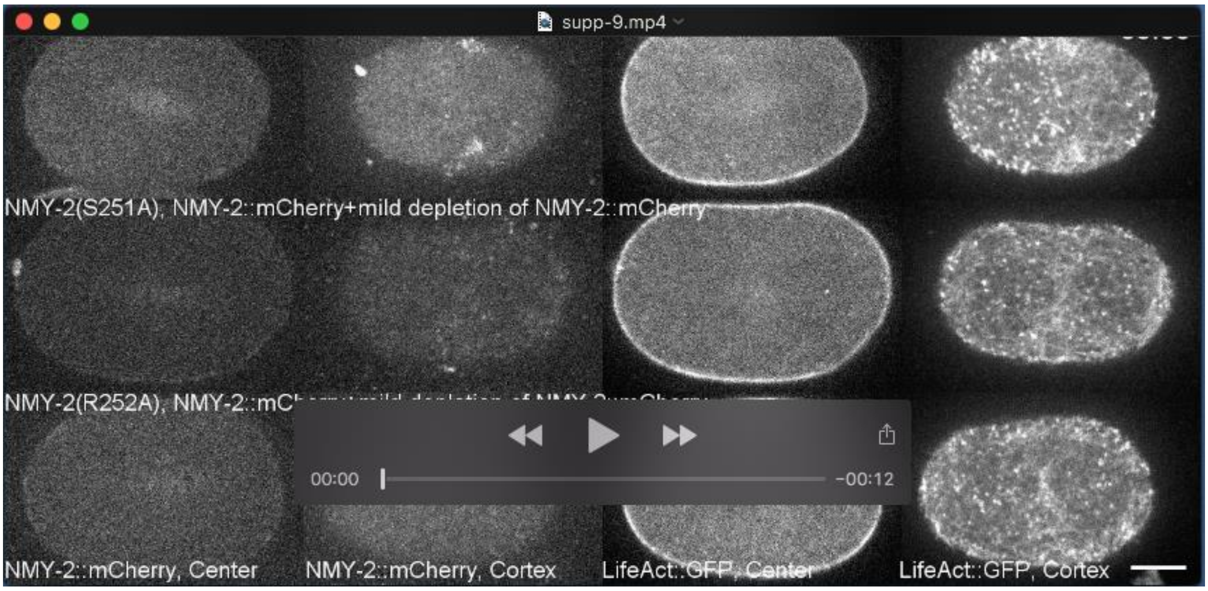

Movie 4. Excess of motor-dead myosin over wild-type myosin results in enhanced recruitment of NMY-2::mCherry^{sen} to the cell equator

Time-lapse images of the central plane (1st and 3rd columns) and cell cortex (2nd and 4th columns) of dividing one-cell embryos expressing NMY-2::mCherry^{sen} (1st and 2nd columns) and LifeAct::GFP (3rd and 4th columns). Conditions are indicated for each row. Cortical images are maximum projections of seven z-sections 0.5 μm apart. First time point corresponds to anaphase onset (0:00) and frames are 5 seconds apart. Frame rate 4 fps, timestamps min:sec. Scale bar, 10 μm.

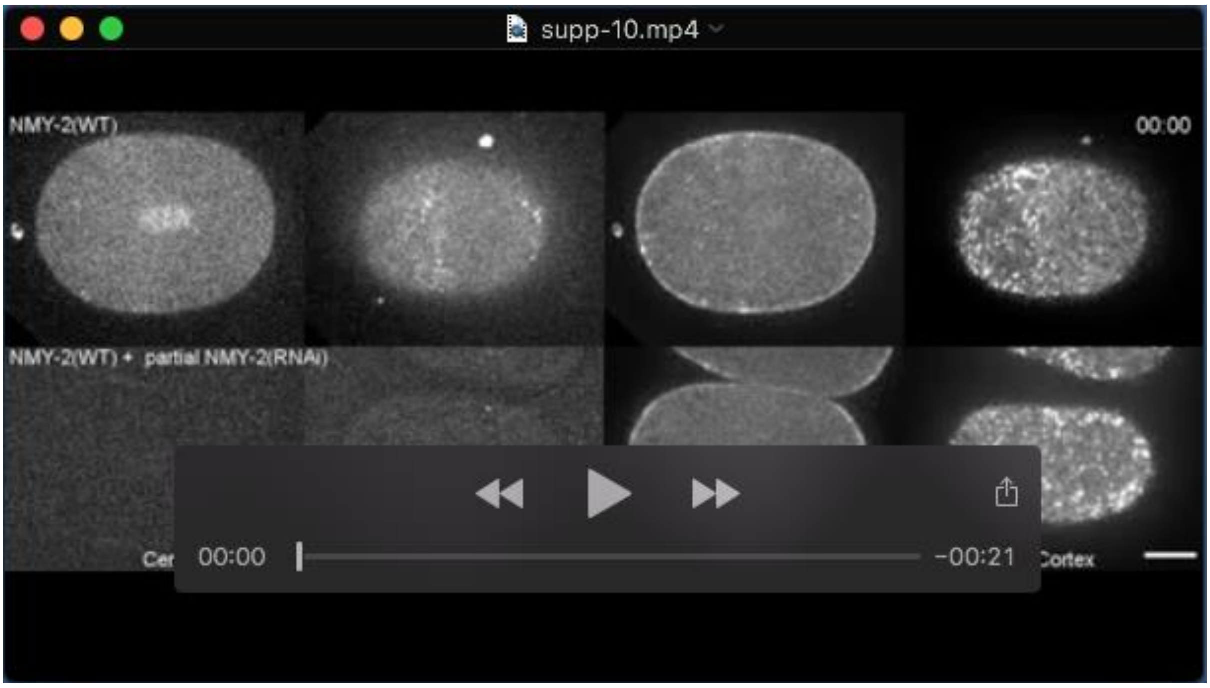

Movie 5. A minimal amount of myosin is required for equatorial deformation in embryos substantially depleted of NMY-2

Time-lapse images of the central plane (1st and 3rd columns) and cell cortex (2nd and 4th columns) of dividing one-cell embryos expressing NMY-2::mCherry^{sen} (1st and 2nd columns) and LifeAct::GFP (3rd and 4th columns). Conditions are indicated for each row. Cortical images are maximum projections of seven z-sections 0.5 µm apart. First time point corresponds to anaphase onset (0:00) and frames are 5 seconds apart. Frame rate 4 fps, timestamps min:sec. Scale bar, 10 µm.

Supplemental Tables

Table S1. List of *C. elegans* strains used in this study

Strain	Genotype	Source/ reference
N2	Ancestral	
EG6429	oxSi36[unc-47::GFP unc-119(+)]IV; unc-41(e268)V	Jorgensen Lab (Frøkjaer-Jensen et al., 2008)
JK2739	mcm-4(e1466) dpy-5(e61) I/hT2 [bli-4(e937) let-?(q782) qIs48] (I;III)	CGC
GCP21	ltIs157 [pAC16;Ppie-1::Life-Act::GFP; unc-119 (+)]; ltIs37 [pAA64; Ppie-1::mCherry::his-58; unc-119 (+)] IV	Our Lab (Silva et al., 2016)
GCP22	ltIs157 [pAC16;Ppie-1::Life-Act::GFP; unc-119 (+)]; unc-119(ed3)III; prtSi2[pAC71; Pnmy-2:: re-encoded nmy-2::mCherry::StrepTagII::3'UTRnmy-2; cb-unc-119(+)]II	This study
GCP179	nmy-2(cp13[nmy-2::GFP + LoxP] I; ltIs44 [pAA173; Ppie-1::mCherry::PH(PLC1delta1); unc-119 (+)]	This study
GCP401	nmy-2 [prt38(S250A)]I; ltIs157 [pAC16;Ppie-1::Life-Act::GFP; unc-119 (+)]; ltIs37 [pAA64; Ppie-1::mCherry::his-58; unc-119 (+)] IV	This study
GCP420	nmy-2 [prt37(R718C)]I; ltIs157 [pAC16;Ppie-1::Life-Act::GFP; unc-119 (+)]; ltIs37 [pAA64; Ppie-1::mCherry::his-58; unc-119 (+)] IV	This study
GCP513	nmy-2 [prt100(R252A)]I/hT2 [bli-4(e937) let-?(q782) qIs48] (I;III).	This study
GCP523	unc-54[prt98(S239A)]I	This study
GCP524	unc-54[prt97(R710C)]I	This study
GCP565	unc-54[prt99(R241A)]I	This study

Development • Supplementary information

GCP592	nmy-2 [prt100(R252A)]I; ltIs157 [pAC16;Ppie-1::Life-Act::GFP; unc-119 (+)]; unc-119(ed3)III; prtSi2[pAC71; Pnmy-2:: re-encoded nmy-2::mCherry::StrepTagII::3'UTRnmy-2; cb-unc-119(+)]II	This study
GCP618	nmy-2 [prt113(S251A)]I; ltIs157 [pAC16;Ppie-1::Life-Act::GFP; unc-119 (+)]; unc-119(ed3)III; prtSi2[pAC71; Pnmy-2::re-encoded nmy-2::mCherry::StrepTagII::3'UTRnmy-2; cb-unc-119(+)]II	This study
GCP619	unc-54[prt112(S240A)]I	This study
GCP629	nmy-2 [prt113(S251A)]I/hT2 [bli-4(e937) let-?(q782) qIs48] (I;III)	This study

Development • Supplementary information

Table S2. List of CRISPR/Cas9 single guide RNAs (sgRNAs) and repair templates used in this study

Gene/Mutation	Repair template*	Diagnosis PCR and restriction enzyme	sgRNA sequence
nmy-2(S250A)	aaccggatcatgggccagcttgaggaacagcttttgcaagcaaatcccatTctTgaAgcAttTggaaacagtaagacagtgaagaacgataatGcTagCagatttgtgagtgatcataactttttaaatgcacgcatcgttaaattttt	Forward primer:<br>ACTCTGGCTTGTTCTGCGTT<br>Reverse primer:<br>GCACGCGAGATTTCTCCAG<br>Restriction enzyme:<br>NheI	sgRNA#1<br>CAAGCAAATCCCATACTCG<br><br>sgRNA#2<br>ATCCCATACTCGAGGCTTT<br><br>sgRNA#3<br>TGATCACTCACAAATCTAC
nmy-2(S251A)	aaccggatcatgggccagcttgaggaacagcttttgcaagcaaatcccatTctTgaAgcAttTggaaacagtaagacagtgaagaacgataatAGCGCTagatttgtgagtgatcataactttttaaatgcacgcatcgttaaattttt	Forward primer:<br>ACTCTGGCTTGTTCTGCGTT<br>Reverse primer:<br>GCACGCGAGATTTCTCCAG<br>Restriction enzyme:<br>Eco47III	
nmy-2(R252A)	aaccggatcatgggccagcttgaggaacagcttttgcaagcaaatcccatTctTgaAgcAttTggaaacagtaagacagtgaagaacgataattcTagCGCTtttgtgagtgatcataactttttaaatgc	Forward primer:<br>ACTCTGGCTTGTTCTGCGTT<br>Reverse primer:<br>GCACGCGAGATTTCTCCAG<br>Restriction enzyme:<br>Eco47III	

Development • Supplementary information

nmy-2(R718C)	cacgaaaagaaacatggagttctcaacgctcatcttgttcttgatcaattGagatgcaacggagtgttAgaaggaattTGCatatgTcgtcaaggattccctacgcggctcccgttccaagaattccgtcaacgcta	Forward primer:<br>TCATTTGTTCAACGCCTCAA<br>Reverse primer:<br>cacgacattgagcttggaaagattcaat<br>Restriction enzyme:<br>NdeI	sgRNA#1<br>CCGCGTAGGGAATCCTTGA<br><br>sgRNA#2<br>GAATTCGTATATGCCGTCA<br><br>sgRNA#3<br>TTAAGATGCAACGGAGTGT
unc-54(S239A)	ccttcggtaacgccaagactgtccgtaacaacaacGcGtcAcgCttTggaaagttcatccgtatccacttcaacaagcacgg	Forward primer:<br>AAGACCACGAGAACCAGTCT<br>Reverse primer:<br>CGTACATGTTGGATGCCTTC<br>Restriction enzyme:<br>MluI	sgRNA#1<br>ACAACAACTCTTCCCGTTT<br><br>sgRNA#2<br>CGGATGAACTTTCCGAAAC<br><br>sgRNA#3<br>ACGGATGAACTTTCCGAAA
unc-54(S240A)	ccttcggtaacgccaagactgtccgtaacaacaacAGCGcTcgtttcggaaagttcatccgtatccacttcaacaa	Forward primer:<br>AAGACCACGAGAACCAGTCT<br>Reverse primer:<br>CGTACATGTTGGATGCCTTC<br>Restriction enzyme:<br>Eco47III	
unc-54(R241A)	tcggtaacgccaagactgtccgtaacaacaactcaAGcGCtttTggaaagttcatccgtatccacttcaacaagcacgg	Forward primer:<br>AAGACCACGAGAACCAGTCT<br>Reverse primer:<br>CGTACATGTTGGATGCCTTC<br>Restriction enzyme:<br>Eco47III	

Development • Supplementary information

unc-54(R710C)	ctttggttctcaaccagcttacctgcaacggagtCCtCgaGggCatATgTatttgcCgTaaAggattccccaacagaacccttcatccagacttcgt	Forward primer:<br>CGAGAAGAACAAGGACCCCC<br>Reverse primer:<br>TGCGAAGCTTCTCCTCCTTG<br>Restriction enzyme:<br>NdeI	sgRNA#1<br>GAATCAGAATTTGCAGAAA<br><br>sgRNA#2<br>GGAATCAGAATTTGCAGAA<br><br>sgRNA#3<br>CCTGCAACGGAGTGTTGGA

*Bases in uppercase indicate silent mutations introduced to avoid repair template recognition by Cas9 and/or to introduce a restriction site (underlined bases) for diagnostic PCR of genomic edits.

Development • Supplementary information

Table S3. List of dsRNAs used in this study

Name	Gene target	Forward primer	Reverse primer	RNAi
nmy-2_RNA#1	nmy-2 (does not target re-encoded nmy-2:mCherry^{sen})	GGCCCGATATCATGAACAACGAGCTTGAAAG	GGCACGATATCAGCCTCCTGGATAGCC	feeding
nmy-2_RNA#2	re-encoded nmy-2:mCherry^{sen} (does not target endogenous nmy-2)	GGCCCGATATCATGAATAATGAACTCGAGTCAATC	GGCCCGATATCACGTTCTTGAATGGCC	feeding
nmy-2_RNA#3	nmy-2 and re-encoded nmy-2:mCherry^{sen}	CCCAAGATATCAATTGAATCTCGGTTGAAGGAA	CCCCCGATATCGACTGCATTTCACGCATCTTATG	feeding
myo-3	myo-3	-------------------	--------------------	feeding (Ahringer library)
unc-54	unc-54	-------------------	--------------------	feeding (Ahringer library)

Development • Supplementary information

Table S4. Primers used in this study

Sequence	Target (gene/plasmid)	Purpose
**Forward**: GAGCTCACCTAGCTAGAAGTCTTT **Reverse**: CGAGATGATGTCATTATTACCGCTGG	nmy-2 (gDNA)	pCFJ151-Pnmy2 5.2kb:: re-encoded nmy2::mCherryStrepTagII::3'nmy2 1.2kb (pAC71)<br><br>nmy-2 promoter fragment
**Forward**: CCAGCGGTAATAATGACATCATCTCG **Reverse**: CCCTTTGAGACCATCTGCAGGTTGC	nmy-2 (pAC65)	pCFJ151-Pnmy2 5.2kb::re-encoded nmy2::mCherryStrepTagII::3'nmy2 1.2kb (pAC71)<br><br>nmy-2 open reading frame fragment
**Forward**: CCCTTTGAGACCATCTGCAGGTTGCG **Reverse**: CCGTACGTCTCGAGTCTAGAGGAATATC	nmy-2 (pAC65)	pCFJ151-Pnmy2 5.2kb::re-encoded nmy2::mCherryStrepTagII::3'nmy2 1.2kb (pAC71)<br><br>nmy-2 3'UTR fragment
**Forward**: CGCAACCTGCAGATGGTCTCAAAGGG **Reverse**: CCCTTTGAGACCATCTGCAGGTTGCG	mCherry (pDC122)	pCFJ151-Pnmy2 5.2kb:: re-encoded nmy2::mCherryStrepTagII::3'nmy2 1.2kb (pAC71)<br><br>mCherry fragment
**Forward:** AAGCTTGTCGAGAAGTACTAGAGGATCATAATC **Reverse:** GCCGCTACCAGAGCCATGG	pACEbac1	pACEbac1-6xHis::nmy2 (1-854) (pAC429)<br><br>backbone+6xHis tag fragment

Development • Supplementary information

**Forward:** ACCATGGCTCTGGTAGCGGCACATCATCTCGACAAAAAGATGATGAG **Reverse:** TAGTACTTCTCGACAAGCTTTTAGTTGCGAACTGAGTCGCGGTCT	nmy-2 (cDNA)	pACEbac1-6xHis::nmy2 (1-854) (pAC429)<br><br>nmy-2 ORF fragment
**Forward:** ACCATGGCTCTGGTAGCGGCACATCATCTCGACAAAAAGATGATGAG **Reverse:** TTAATCTTCTTCGAGCTGACGAATTT	nmy-2 (cDNA)	pACEbac1-6xHis::nmy2 (1-1354) (pAC514)<br><br>nmy-2 1-1354 ORF fragment
**Forward:** AAGCTTGTCGAGAAGTACTAGAGGATCATAATC **Reverse:** GCCGCTACCAGAGCCTTTTTC	pACEbac1	pACEbac1-StrepTagII::mlc-4 (pAC437)<br>pACEbac1-StrepTagII::mlc-5 (pAC438)<br><br>backbone+StrepTagII fragment
**Forward:** AAAAAGGCTCTGGTAGCGGCGCCTCCCGCAAAACCGTAAAC **Reverse:** TAGTACTTCTCGACAAGCTTTTAAGCCTCATCCTTGTCCTTGG	mlc-4 (cDNA)	pACEbac1-StrepTagII::mlc-4 (pAC437)<br><br>mlc-4 ORF fragment
**Forward:** AAAAAGGCTCTGGTAGCGGCGACGATTTGGCTGATTGTCGTG **Reverse:** AGTACTTCTCGACAAGCTTTTAGGAGTTCATGACAGCGCG	mlc-5 (cDNA)	pACEbac1-StrepTagII::mlc-5 (pAC438)<br><br>mlc-5 ORF fragment
**Forward:** AAAAAGGCTCTGGTAGCGGCGAGCAGGATGAGCTGCGTG **Reverse:** TAGTACTTCTCGACAAGCTTACTATTGATAGATTGTGGAAGAG	let-502 (cDNA)	pACEbac1-StrepTagII::let-502 (pAC522)<br><br>let-502 full length ORF fragment

Development • Supplementary information

**Forward:** TAGTAAGCTTGTCGAGAAGTAC **Reverse:** TTCGAATTCTCGGTTTTTC	pACEbac1-StrepTagII::LET-502 (pAC522)	pACEbac1-StrepTagII::let-502 (1-469)(pAC527)
**Forward:** GACAGTGAAGAACGATAATGCCA GTAGATTTGGAAAGTTC **Reverse:** GAACTTTCCAAATCTACTGGCATT ATCGTTCTTCACTGTC	pACEbac1-6xhis::NMY-2 (1-854) (pAC429)<br><br>pACEbac1-6xHis::NMY-2 (1-1354) (pAC514)	pACEbac1-6xHis::nmy-2 (S250A) (1-854) (pAC451)<br><br>pACEbac1-6xHis::nmy2 (S250A)(1-1354) (pAC515)
**Forward:** CAGTGAAGAACGATAATTCCGCT AGATTTGGAAAGTTCATTC **Reverse:** GAATGAACTTTCCAAATCTAGCG GAATTATCGTTCTTCACTG	pACEbac1-6xHis::NMY-2 (1-854) (pAC429)<br><br>pACEbac1-6xHis::NMY-2 (1-1354) (pAC514)	pACEbac1-6xHis::nmy2 (S251A)(1-854) (pAC453)<br><br>pACEbac1-6xHis::nmy2 (S251A)(1-1354) (pAC516)
**Forward:** GTGAAGAACGATAATTCCAGTGC ATTTGGAAAGTTCATTCGCG **Reverse:** CGCGAATGAACTTTCCAAATGCA CTGGAATTATCGTTCTTCAC	pACEbac1-6xHis::NMY-2 (1-854) (pAC429)<br>pACEbac1-6xHis::NMY-2 (1-1354) (pAC514)	pACEbac1-6xHis::nmy2 (R252A)(1-854) (pAC452)<br>pACEbac1-6xHis::nmy2 (R252A)(1-1354) (pAC517)
**Forward:** CGGAGTGTTGGAAGGAATTTGTA TATGCCGTCAAGGATTC **Reverse:** GAATCCTTGACGGCATATACAAA TTCCTTCCAACACTCCG	pACEbac1-6xHis::NMY-2 (1-854) (pAC429)	pACEbac1-6xHis::nmy2 (R781C)(1-854) (pAC476)

Development • Supplementary information

Table S5. Plasmids used in this study

Plasmid	Source	Reference	Use
Peft-3::Cas9 + Empty sgRNA	Addgene	RRID:Addgene_47549	Expression of CAS-9 and guide RNAs
pCFJ151	Addgene	RRID:Addgene_19330	Generation of MosSCI strains
pCFJ601	Addgene	RRID:Addgene_34874	MosSCI strains co-injection markers
pCFJ90	Addgene	RRID:Addgene_19327	MosSCI strains co-injection markers
pCFJ104	Addgene	RRID:Addgene_19328	MosSCI strains co-injection markers
pGH8	Addgene	RRID:Addgene_19359	MosSCI strains co-injection markers
L4440	Addgene	RRID:Addgene_1654	Plasmid for RNAi by feeding
pCFJ151-Pnmy2 5.2kb::nmy2 reenc::mCherryStrepTagII::3'nmy2 1.2kb	This study	pAC71	Plasmid for generation of NMY-2::mCherry^{sen} (GCP22)
pACEBac1-6xHis::nmy-2(1-874)_WT	This study	pAC429	Plasmid for Baculovirus expression 6xHis NMY-2$_{S1}$
pACEBac1-6xHis::nmy-2(1-874)_S250A	This study	pAC451	Plasmid for Baculovirus expression 6xHis NMY-2(S250A)$_{S1}$
pACEBac1-6xHis::nmy-2(1-874)_S251A	This study	pAC453	Plasmid for Baculovirus expression 6xHis NMY-2(S251A)$_{S1}$

Development • Supplementary information

pACEBac1-6xHis::nmy-2(1-874)_R252A	This study	pAC452	Plasmid for Baculovirus expression 6xHis NMY-2(R252A)$_{S1}$
pACEBac1-6xHis::nmy-2(1-1354)_WT	This study	pAC514	Plasmid for Baculovirus expression 6xHis NMY-2$_{HMM}$
pACEBac1-6xHis::nmy-2(1-1354)_S250A	This study	pAC515	Plasmid for Baculovirus expression 6xHis NMY-2(S250A)$_{HMM}$
pACEBac1-6xHis::nmy-2(1-1354)_S251A	This study	pAC516	Plasmid for Baculovirus expression 6xHis NMY-2(S251A)$_{HMM}$
pACEBac1-6xHis::nmy-2(1-1354)_R252A	This study	pAC517	Plasmid for Baculovirus expression 6xHis NMY-2(R252A)$_{HMM}$
pACEBac1-StrepTagII::mlc-4	This study	pAC437	Plasmid for Baculovirus expression StrepTagII MLC-4
pACEBac1-StrepTagII::mlc-5	This study	pAC438	Plasmid for Baculovirus expression StrepTagII MLC-5
pACEBac1-StrepTagII::Let-502(1-469)	This study	pAC527	Plasmid for Baculovirus expression StrepTagII LET-502(1-469)

Development • Supplementary information

Supplemental References

Frøkjær-Jensen, C., Davis, M. W., Hopkins, C. E., Newman, B. J., Thummel, J. M., Olesen, S.-P., Grunnet, M. and Jorgensen, E. M. (2008). Single-copy insertion of transgenes in Caenorhabditis elegans. *Nat. Genet*. **40**, 1375–1383. doi:10.1038/ng.248

Silva, A. M., Osório, D. S., Pereira, A. J., Maiato, H., Pinto, I. M., Rubinstein, B., Gassmann, R., Telley, I. A. and Carvalho, A. X. (2016). Robust gap repair in the contractile ring ensures timely completion of cytokinesis. *J. Cell Biol.* **215**, 789-799. doi:10.1083/jcb.201605080

Development • Supplementary information
